# HMGB1 orchestrates tumor-osteoclast crosstalk to drive bone metastasis in hepatocellular carcinoma

**DOI:** 10.1038/s41419-025-08037-6

**Published:** 2025-10-07

**Authors:** Yan-zhu Chen, Di Xu, Ya-xun Jia, Jie Ma, Zuo-lin Xiang

**Affiliations:** 1 Department of Radiation Oncology, Shanghai East Hospital, School of Medicine, Tongji University, Shanghai, China; 2 Department of Radiation Oncology, Shanghai East Hospital Ji’an Hospital (Ji’an Central People’s Hospital), Ji’an, China

**Keywords:** Bone metastases, Bone cancer

## Abstract

Bone metastasis in hepatocellular carcinoma (HCC) poses a significant clinical challenge, characterized by poor prognosis and severe skeletal complications. This study identifies the HMGB1/LCN2/JAK1/STAT3 axis as the central mechanism driving HCC bone metastasis through tumor-osteoclast crosstalk. High-mobility group box 1 (HMGB1) induces osteoclast activation and differentiation, promoting lipocalin-2 (LCN2) secretion by osteoclasts, which activates the JAK1/STAT3 pathway in HCC cells, forming a feedback loop that enhances osteolytic bone resorption and tumor dissemination. Integrated single-cell and bulk RNA sequencing reveal enriched osteoclast-related and pro-metastatic pathways in the tumor-bone microenvironment, while functional assays involving knockdown and overexpression demonstrate that modulating the HMGB1/LCN2/JAK1/STAT3 axis regulates osteoclast activity, tumor growth, and bone destruction in vitro and in vivo. These results suggest the HMGB1/LCN2/JAK1/STAT3 axis as a potential therapeutic target, offering a strategy to reduce skeletal damage and systemic tumor progression, thereby contributing to improved management of advanced HCC.

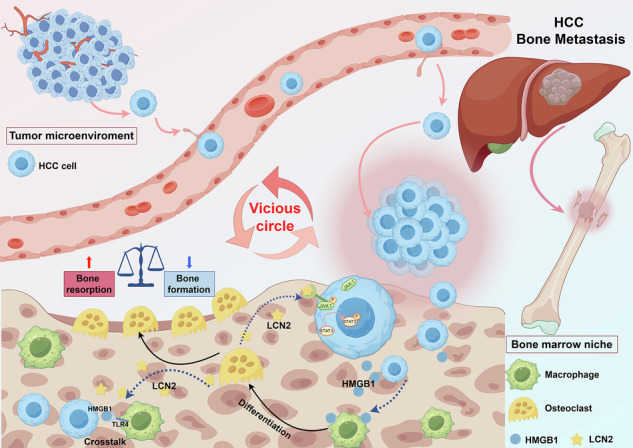

## Introduction

Hepatocellular carcinoma (HCC) ranks as the third leading cause of cancer-related mortality worldwide, with a high propensity for distant metastasis [1]. Among metastatic sites, the skeleton represents the second most common target, with bone metastasis affecting 25.5–38.5% of patients [2, 3]. These patients experience a poor prognosis, with a median survival of only 4.6 months following diagnosis, accompanied by significant complications such as severe pain, fractures, and neurological compression [4, 5]. Thus, a deeper understanding of the mechanisms governing the tumor-bone microenvironment is essential for developing effective therapies targeting metastasis with clinical relevance.

The bone-tumor microenvironment is a complex and dynamic system comprising tumor cells, osteoclasts, stromal cells, and immune components that interact through intricate signaling networks to drive tumor progression [6]. In HCC, osteolytic bone destruction is the predominant phenotype of bone metastasis, primarily mediated by excessive osteoclast activation [7]. Tumor-derived factors such as parathyroid hormone-related protein (PTHrP), interleukin-6 (IL-6), and tumor necrosis factor-alpha (TNF-α), have been shown to promote osteoclast differentiation and activity, thereby accelerating bone resorption [7, 8]. This resorptive process, in turn, releases matrix-embedded growth factors, including transforming growth factor-beta (TGF-β), insulin-like growth factor (IGF), and calcium, which further enhance tumor cell proliferation, survival, and invasion [8, 9]. Notably, the crosstalk between osteoclasts and tumor cells establishes a vicious cycle that sustains bone destruction and tumor growth [10]. Emerging evidence from other malignancies, such as breast and lung cancers, underscores the multifaceted roles of osteoclasts beyond bone resorption [11, 12]. For instance, osteoclasts have been shown to suppress T cell-mediated immunity and secrete pro-tumorigenic factors, actively contributing to tumor progression within the bone microenvironment [13]. Together, the osteolytic phenotype and tumor-osteoclast interactions play pivotal roles in HCC bone metastasis, emphasizing the need for targeted strategies to interrupt this pathological cycle and improve clinical outcomes.

High-mobility group box 1 (HMGB1), a widely expressed chromatin-associated protein, regulates nuclear functions such as gene transcription, DNA repair, and chromatin remodeling [14], modulating processes such as cell differentiation, migration, inflammation, and tumor progression [15, 16]. HMGB1’s oncogenic roles are well-documented in cancers such as colorectal, oral, and breast cancers [17–19], with growing evidence of its importance in HCC. In HCC, HMGB1 promotes tumor growth through immune modulation [20] and enhances metastatic potential under hypoxic conditions via mechanisms involving mitochondrial dynamics [21]. Within the bone microenvironment, HMGB1 interacts with toll-like receptor 4 (TLR4) to regulate osteoclast activation and modulate macrophage responses, processes implicated in osteolytic bone destruction [22–24]. However, its specific role in the HCC tumor-bone microenvironment, particularly in orchestrating tumor-osteoclast interactions to promote osteolytic bone metastasis, remains underexplored. This gap highlights the need for detailed investigation to clarify HMGB1’s contribution to HCC bone metastasis and to identify potential therapeutic targets.

This study aims to investigate the role of HMGB1 in facilitating tumor progression, with a particular emphasis on its interactions with osteoclast activation and the JAK1/STAT3 signaling pathway within the tumor-bone microenvironment, providing a foundation for understanding the mechanisms of HCC bone metastasis.

## Results

### HMGB1 is an unfavorable prognostic factor and driver of bone metastasis in HCC

**Fig. 1:**
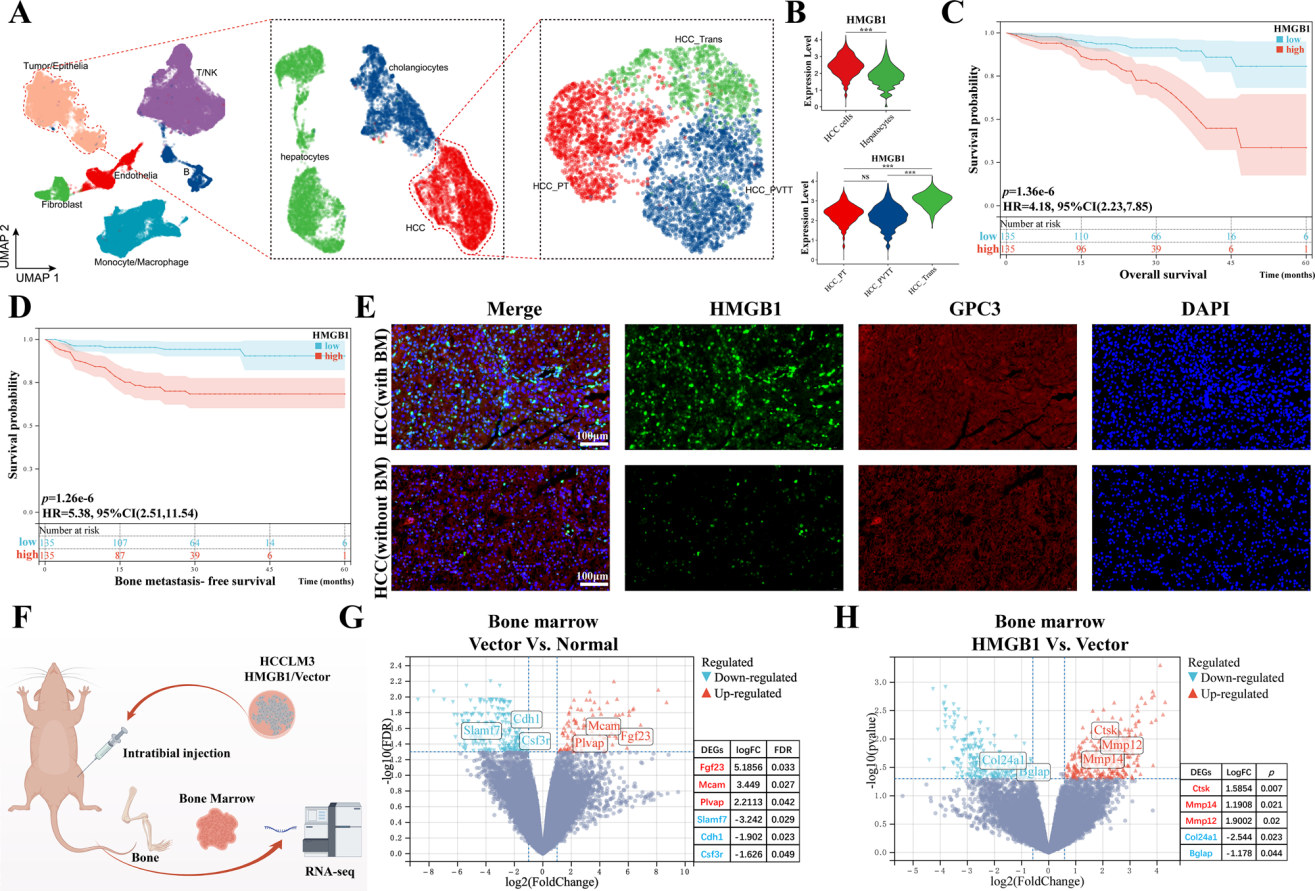
HMGB1 expression and its role in HCC bone metastasis progression. **A** UMAP of single-cell RNA-seq data identifying three HCC subpopulations: primary tumor group (HCC^PT^), transformation group with metastatic potential (HCC^Trans^), and metastatic lesion group (HCC^PVTT^). **B** Violin plots showing HMGB1 upregulation in HCC cells (*p* < 0.001), particularly in the HCC^Trans^ subpopulation (*p* < 0.001). **C**, **D** Kaplan–Meier curve depicting OS and BM-FS of HCC patients stratified by high and low HMGB1 expression. **E** Representative immunofluorescence images of HMGB1 expression in HCC tissues with or without bone metastasis. **F** Schematic of the HCC bone metastasis mouse model and bone marrow RNA-seq analysis. **G** Volcano plot of differentially expressed genes in bone marrow (Vector vs. Normal) showing upregulation of osteoclast-related genes and downregulation of epithelial and immune markers. **H** Volcano plot of differentially expressed genes in bone marrow (HMGB1 vs. Vector) highlighting osteoclast-associated genes and osteogenesis-related markers.

To explore the role of HMGB1 in HCC metastasis, a metastatic HCC single-cell RNA sequencing (scRNA-seq) dataset was analyzed. As described in our previous studies [25], UMAP-based dimensionality reduction identified three HCC subpopulations: the primary tumor group (HCC^PT^), the transformation group with metastatic potential (HCC^Trans^), and the metastatic lesion group (HCC^PVTT^) (Fig. 1A). HMGB1 expression was significantly upregulated in HCC cells compared to normal hepatocytes, with the highest expression observed in the HCC^Trans^ subpopulation (Fig. 1B). To assess clinical relevance, HMGB1 protein expression was assessed in tissue microarrays from a cohort of 270 HCC patients at Shanghai East Hospital using immunohistochemistry. Staining intensities, quantified with QuPath software, enabled stratification of patients into high and low HMGB1 expression groups based on median values. Statistical analysis revealed significant associations between high HMGB1 expression and increased bone metastasis (*p* = 2.7e−6), larger tumor size (*p* = 7.0e−7), advanced BCLC stage (*p* = 2.5e−10), and vascular invasion (*p* = 2.5e−4) (Table 1). Survival analysis further indicated that patients with high HMGB1 expression experienced reduced overall survival (OS, Fig. 1C) and bone metastasis-free survival (BM-FS, Fig. 1D). Immunofluorescence analysis confirmed elevated HMGB1 protein expression in tumor tissues from bone metastasis patients compared to non-metastasis controls (Fig. 1E).

**Table 1:** Demographic and clinical characteristics of patients included in this study.

		HMGB1 expression group		*p* value
		High (*n* = 135)	Low (*n* = 135)	
Age (years)				6.9e−4
	Mean ± SD	62.05 ± 7.96	65.43 ± 8.20	
Gender				0.87
	Male	113 (41.85%)	111 (41.11%)	
	Female	22 (8.15%)	24 (8.89%)	
Bone metastasis				2.7e−6
	Yes	38 (14.07%)	8 (2.96%)	
	No	97 (35.93%)	127 (47.04%)	
HBsAg				0.11
	Positive	102 (37.78%)	89 (32.96%)	
	Negative	33 (12.22%)	46 (17.04%)	
AFP (ng/ml)				0.30
	≤20	87 (32.22%)	96 (35.56%)	
	>20	48 (17.78%)	39 (14.44%)	
BCLC stage				2.5e−10
	0–A	42 (15.56%)	95 (35.19%)	
	B–C	93 (34.44%)	40 (14.81%)	
Tumor size (cm)				7.0e−7
	≤5	34 (12.59%)	75 (27.78%)	
	>5	101 (37.41%)	60 (22.22%)	
vascular invasion				2.5e−4
	Yes	79 (29.26%)	48 (17.78%)	
	No	56 (20.74%)	87 (32.22%)	
Intrahepatic control				1.5e−12
	Yes	43 (15.93%)	102 (37.78%)	
	No	92 (34.07%)	33 (12.22%)	

*NA* Not applicable.

To further investigate the functional role of HMGB1, a mouse model of HCC bone metastasis was established (Fig. 1F). RNA-seq of bone marrow from this model identified significant changes in gene expression. Compared to the normal group, 122 genes were upregulated in the Vector group, including osteoclast-related Fgf23, the poor prognostic marker Mcam, and angiogenesis-related Plvap, alongside downregulation of 246 genes, such as the epithelial marker Cdh1 and immune-related genes Slamf7 and Csf3r, were downregulated (Fig. 1G). Gene Set Enrichment Analysis (GSEA) identified enrichment of the “GOBP_lymphocyte activation involved in immune response” pathway in the normal group, indicating enhanced anti-tumor immunity (Supplementary Fig. S1A), while the “HALLMARK_epithelial mesenchymal transition” (EMT) pathway was prominent in the Vector group, indicating enhanced metastatic potential (Supplementary Fig. S1B).Further comparison between the HMGB1 group and the Vector group revealed 258 upregulated genes in the HMGB1 group, including osteoclast-associated genes Ctsk, Mmp14, and Mmp12, and 229 downregulated genes, such as osteogenesis-related markers Col24a1 and Bglap (Fig. 1H). GSEA shows that pathways such as “MP_increased metastatic potential” (Supplementary Fig. S1C) and the “HALLMARK_epithelial mesenchymal transition” (Supplementary Fig. S1D) were significantly enriched in the HMGB1 group, reinforcing the pro-metastatic role of HMGB1. Additionally, GSEA analysis demonstrated significant enrichment of the “GOBP bone_resorption” and “GOBP bone_remodeling” pathways in the HMGB1 group (Supplementary Fig. S1E), consistent with increased osteoclast activity.

### HMGB1 drives HCC bone metastasis and induces skeletal complications

**Fig. 2:**
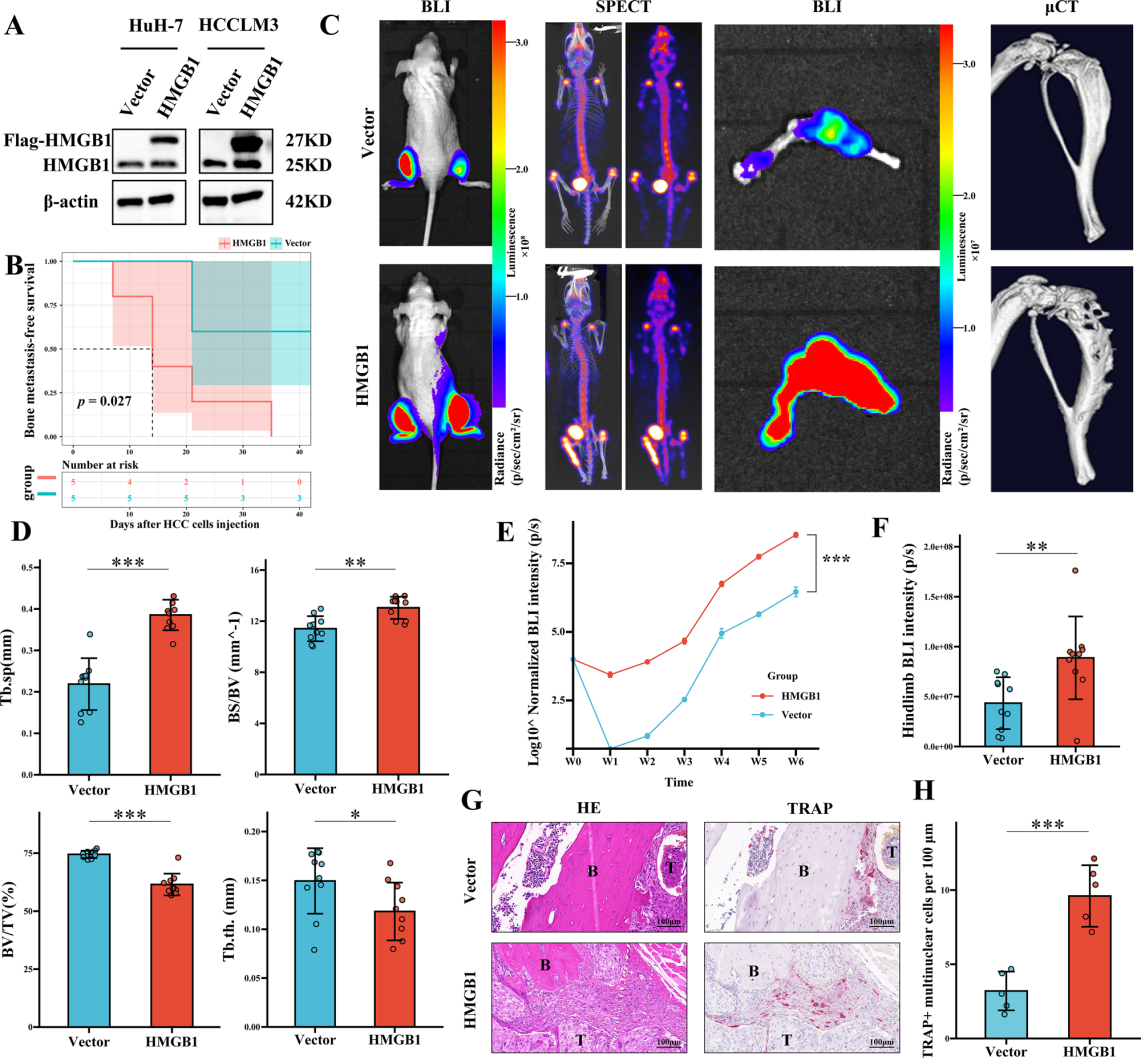
HMGB1 promotes HCC bone metastasis and skeletal complications in vivo. **A** Western blot analysis validating HMGB1 overexpression in HuH-7 and HCCLM3 cells. **B** Kaplan–Meier curve showing earlier onset of bone metastasis in nude mice injected with HMGB1-overexpressing HCCLM3 cells. **C** Representative images from BLI, SPECT, and μCT demonstrating increased systemic bone metastasis and severe osteolytic lesions in the HMGB1-overexpression group compared to the control. **D** μCT quantification of bone parameters, showing increased Tb.sp and BS/BV, alongside decreased BV/TV and Tb.th in the HMGB1-overexpression group. **E** In vivo BLI at multiple time points, quantifying tumor burden based on signal intensity in mice. **F** Ex vivo BLI of hind limbs at the experimental endpoint, assessing tumor burden based on signal intensity in mice. **G** HE staining depicting osteolytic bone destruction and TRAP positive osteoclast areas following HMGB1 overexpression. **H** Quantitative analysis of TRAP-positive osteoclast areas, indicating increased osteoclast activation in the HMGB1-overexpression group.

To assess the functional role of HMGB1 in promoting HCC bone metastasis, stable cell lines with HMGB1 overexpression (Fig. 2A) and knockdown (Supplementary Fig. S2A) were established in HuH-7 and HCCLM3 cells. Intratibial injection of these cells into nude mice was performed, followed by weekly monitoring of bone metastasis progression using bioluminescence imaging (BLI).

Mice injected with HMGB1-overexpressing cells showed significantly earlier onset of systemic bone metastasis (Fig. 2B), BLI, single-photon emission computed tomography (SPECT), and micro-computed tomography (μCT) analyses indicated an increased tumor burden and skeletal-related events (SREs) in the HMGB1 overexpression group compared to controls (Fig. 2C). Quantitative μCT analysis revealed severe osteolytic lesions and a higher incidence of skeletal-related events (SREs), characterized by increased trabecular separation (Tb.sp) and bone surface-to-volume ratio (BS/BV), alongside decreased bone volume fraction (BV/TV) and trabecular thickness (Tb.th). (Fig. 2D). In vivo BLI at multiple time points (Fig. 2E) and Ex vivo BLI of hind limbs demonstrated an elevated tumor burden in the HMGB1 overexpression group (Fig. 2F). Histological analysis further confirmed larger osteolytic areas and a notable increase in tartrate-resistant acid phosphatase (TRAP)-positive osteoclasts at the bone-tumor interface (Fig. 2G, H), suggesting enhanced osteoclast activation.

In contrast, HMGB1 knockdown in HCCLM3 cells significantly suppressed bone metastasis progression. Mice injected with HMGB1-knockdown cells exhibited reduced tumor burden and fewer SREs compared to controls (Supplementary Fig. S2B). μCT analysis indicated altered bone structural parameters (Fig. S2C), BLI demonstrated HMGB1 knockdown reduce tumor burden (Supplementary Fig. S2D, E), while TRAP staining revealed decreased osteoclast activity at the bone-tumor interface (Supplementary Fig. S2F, G). Together, these results indicate that HMGB1 is associated with HCC bone metastasis and skeletal complications, linked to enhanced osteoclast activation and osteolytic bone destruction.

### HMGB1-mediated osteoclastogenesis and tumor-macrophage interactions in tumor-bone microenvironment

**Fig. 3:**
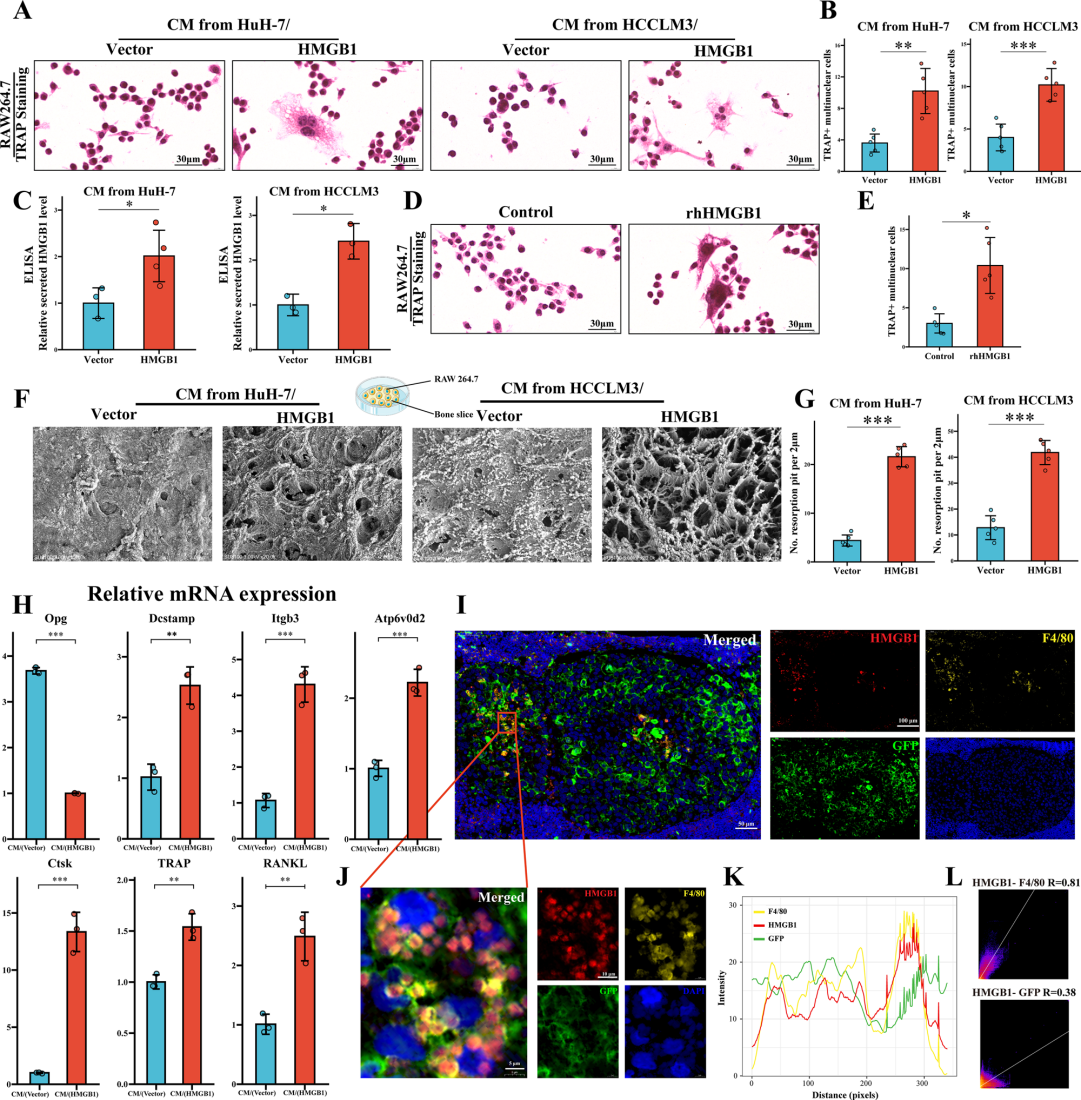
HMGB1 enhances osteoclastogenesis and facilitates interaction of HCC cells and macrophages in the tumor-bone microenvironment. **A** TRAP staining of RAW264.7 cells treated with CM from HMGB1-overexpressing HCC cells. **B** Quantification of TRAP-positive osteoclasts, confirming a significant increase in multinucleated cells treated with CM from HMGB1-overexpressing HCC cells. **C** ELISA analysis demonstrating elevated HMGB1 concentrations in the culture supernatants of HMGB1-overexpressing HCC cells. **D** TRAP staining of RAW264.7 cells treated with recombinant rhHMGB1. **E** Quantification of TRAP-positive osteoclasts, verifying enhanced osteoclast differentiation in RAW264.7 cells treated with rhHMGB1. **F** The scanning electron microscopy image of bone resorption assay showing increased resorption pit formation on bone slices treated with CM from HMGB1-overexpressing HCC cells. **G** Quantification of resorption pits, confirming severe surface erosion on bone slices treated with CM from HMGB1-overexpressing HCC cells. **H** qRT-PCR analysis showing upregulation of osteoclast differentiation and activation markers (TRAP, RANKL, Atp6v0d2, Itgb3, Ctsk, Opg, Dcstamp) in RAW264.7 cells treated with CM from HMGB1-overexpressing HCC cells. **I** mIHC of HCC bone metastasis tissues from the animal model showing HMGB1 (red) co-localized with macrophages (F4/80, yellow) near HCC cells (GFP, green), with DAPI (blue) staining nuclei. **J** Magnified mIHC image highlighting the co-localization of HMGB1 with GFP-labeled HCC cells and F4/80-labeled macrophages in the tumor-bone interface. **K** Quantitative fluorescence intensity analysis along selected lanes in the mIHC overlay images, showing co-localization patterns. **L** Correlation analysis presenting high Pearson’s correlation coefficients that confirm significant co-localization of HMGB1 with GFP-labeled HCC cells (R = 0.38) and F4/80-labeled macrophages (R = 0.81).

To investigate the role of HMGB1 in osteoclastogenesis, conditioned medium (CM, Supplementary File, Materials and Methods for further details) from HMGB1-overexpressing HCC cells was applied to RAW264.7 cells. TRAP staining revealed a significant increase in the number of multinucleated TRAP^+^ osteoclasts treated with CM from HMGB1-overexpressing cells compared to controls (Fig. 3A, B). Enzyme-linked immunosorbent assay (ELISA) confirmed higher HMGB1 concentrations in the culture supernatants of HMGB1-overexpressing HCC cells (Fig. 3C). To isolate the direct effect of HMGB1 on osteoclast differentiation, RAW264.7 cells treated with recombinant human HMGB1 (rhHMGB1) exhibited enhanced osteoclast differentiation (Fig. 3D, E), consistent with the results from CM treatment of HMGB1-overexpressing HCC cells. A bone resorption assay demonstrated severe surface erosion and increased formation of resorption pits in bone slices treated with CM from HMGB1-overexpressing cells (Fig. 3F, G). Moreover, quantitative real-time PCR (qRT-PCR) analysis demonstrated that osteoclasts induced by CM from HMGB1-overexpressing HCC cells exhibited significantly increased expression levels of osteoclast differentiation and activation markers, including TRAP, RANKL, Atp6v0d2, Itgb3, Ctsk, and Dcstamp. In contrast, the expression of Opg, a soluble decoy receptor that inhibits osteoclastogenesis by neutralizing RANKL, was markedly decreased (Fig. 3H). These findings demonstrate that HMGB1 secreted by HCC cells, with elevated levels in CM, contributes to the differentiation of mononuclear progenitor osteoclasts into multinucleated mature osteoclasts.

To investigate cellular interactions within the HCC bone microenvironment, multiplex immunohistochemistry (mIHC) was performed on tissue sections from the HCC bone metastasis mouse model. Figure 3I illustrates the spatial distribution of HMGB1 (red), F4/80-labeled macrophages (yellow), and GFP-labeled HCC cells (green) at the tumor-bone microenvironment. The magnified region of the mIHC images highlighted the complex tumor-bone microenvironment of HCC bone metastasis, showing co-localization of HMGB1 with GFP-labeled HCC cells infiltrating the bone marrow cavity and with F4/80-labeled macrophages in certain areas (Fig. 3J). Quantitative fluorescence intensity analysis along selected lanes in the overlay images revealed co-localization patterns (Fig. 3K). Correlation analysis with the Coloc2 plugin confirmed significant co-localization of HMGB1 with GFP-labeled HCC cells and F4/80-labeled macrophages, as evidenced by high Pearson’s correlation coefficients (Fig. 3L).

### HMGB1-LCN2 mediates crosstalk among HCC cells, macrophages, and osteoclasts in the tumor-bone microenvironment

**Fig. 4:**
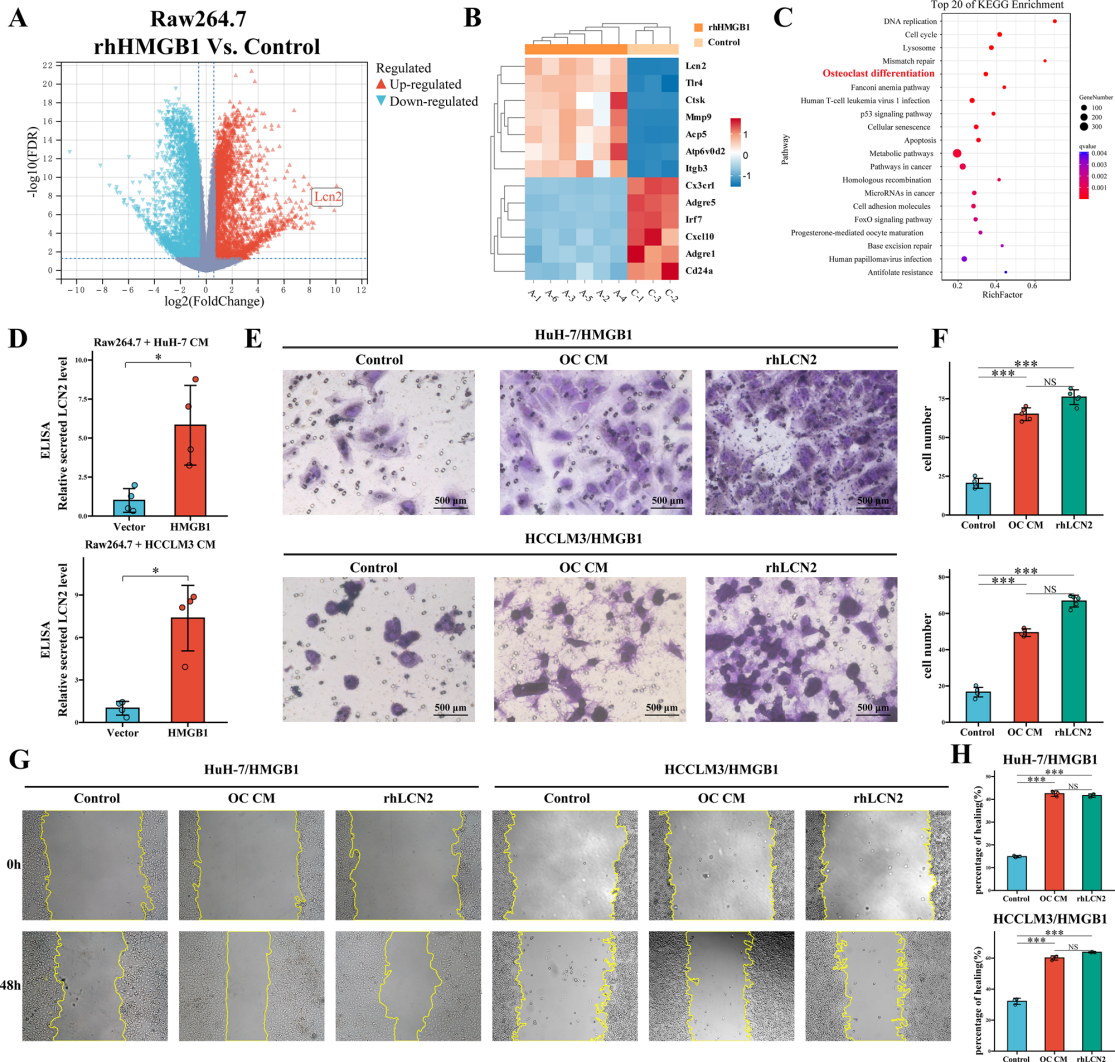
HMGB1 promotes osteoclast differentiation and LCN2 secretion, enhancing tumor invasion. **A** Volcano plot showing significantly upregulated (red) and downregulated (blue) genes in RAW264.7-derived osteoclasts treated with rhHMGB1 compared to controls. **B** Heatmap highlighting representative genes altered by rhHMGB1 treatment, including upregulation of Lcn2, Tlr4, and osteoclast-related genes and downregulation of immune/macrophage markers. **C** KEGG enrichment analysis plot illustrating the significant enrichment of the osteoclast differentiation pathway among differentially expressed genes. **D** ELISA indicating elevated LCN2 secretion by osteoclasts treated with CM from HMGB1-overexpressing HCC cells. **E** Transwell invasion assay images demonstrating enhanced invasive capacity of HMGB1-overexpressing HCC cells treated with OC CM or rhLCN2. **F** Quantification of Transwell invasion assay, confirming the increased invasive ability of HMGB1-overexpressing HCC cells. **G** Wound healing assay images showing increased migratory capacity of HMGB1-overexpressing HCC cells treated with OC CM or rhLCN2. **H** Quantification of wound healing assay, verifying the enhanced migration of HMGB1-overexpressing HCC cells.

To explore the role of HMGB1 in tumor-bone microenvironmental interactions, RNA-seq was performed on RAW264.7-derived osteoclasts under two conditions: a control group (osteoclasts induced under standard conditions) and an rhHMGB1-treated group (osteoclasts induced with 100 ng/mL rhHMGB1 in addition to standard conditions). Differential expression analysis identified 2009 upregulated and 2112 downregulated genes in the rhHMGB1-treated group (Fig. 4A). Upregulated genes included the secretory protein Lcn2, the recognized macrophage activation marker Tlr4, and osteoclast-related genes such as Ctsk, Acp5, and Atp6v0d2, whereas downregulated genes mainly involved immune and macrophage markers, including Adgre1, Adgre5, and Cd24a (Fig. 4B). KEGG pathway enrichment analysis revealed significant enrichment of the osteoclast differentiation pathway (Fig. 4C). GSEA further confirmed enrichment of the “GOBP_osteoclast_differentiation” gene set in the rhHMGB1 group (Supplementary Fig. S3A), alongside the “BROWN_myeloid_cell_development_up” gene set (Supplementary Fig. S3B). Conversely, the “BROWN_myeloid_cell_development_dn” gene set was enriched in the control group (Supplementary Fig. S3C). Additionally, Immunofluorescence staining demonstrated increased expression of TLR4 in RAW264.7 cells following treatment with rhHMGB1 (Supplementary Fig. S4A), supporting the presence of an HMGB1-TLR4 molecular interaction in macrophages within the tumor-bone microenvironment.

Then, ELISA demonstrated significantly elevated LCN2 levels in the supernatants of osteoclasts treated with CM from HMGB1-overexpressing HCC cells (Fig. 4D). To evaluate the impact of osteoclast-derived LCN2 on tumor progression, we co-cultured HCC cells with osteoclast-conditioned medium (OC CM, Supplementary File, Materials and Methods for further details) or recombinant LCN2 (rhLCN2) protein. Transwell invasion assays showed that both OC CM and rhLCN2 significantly enhanced the invasive ability of HMGB1-overexpressing HCC cells (Fig. 4E, F). Similarly, wound healing assays showed increased migration of HMGB1-high HCC cells upon treatment with OC CM or rhLCN2 (Fig. 4G, H). Notably, immunofluorescence analysis confirmed a dose-dependent increase in TLR4 expression in RAW264.7 cells treated with rhLCN2 (Supplementary Fig. S4B). Co-immunoprecipitation (Co-IP) experiments further confirmed the interaction between HMGB1 and TLR4, with rhLCN2 enhancing this interaction in a concentration-dependent manner (Supplementary Fig. S4C, D).

In summary, these findings establish a HMGB1-LCN2 feedback loop within the tumor-bone microenvironment: HMGB1 derived from HCC cells promotes osteoclast differentiation and LCN2 secretion, while osteoclast-secreted LCN2 enhances HCC cell invasion and migration and strengthens the HMGB1-TLR4 interaction in macrophages. This crosstalk reinforces osteoclastogenesis and drives tumor progression.

### Osteoclast-derived LCN2 activates the JAK1/STAT3 pathway in HMGB1-overexpressing HCC cells

**Fig. 5:**
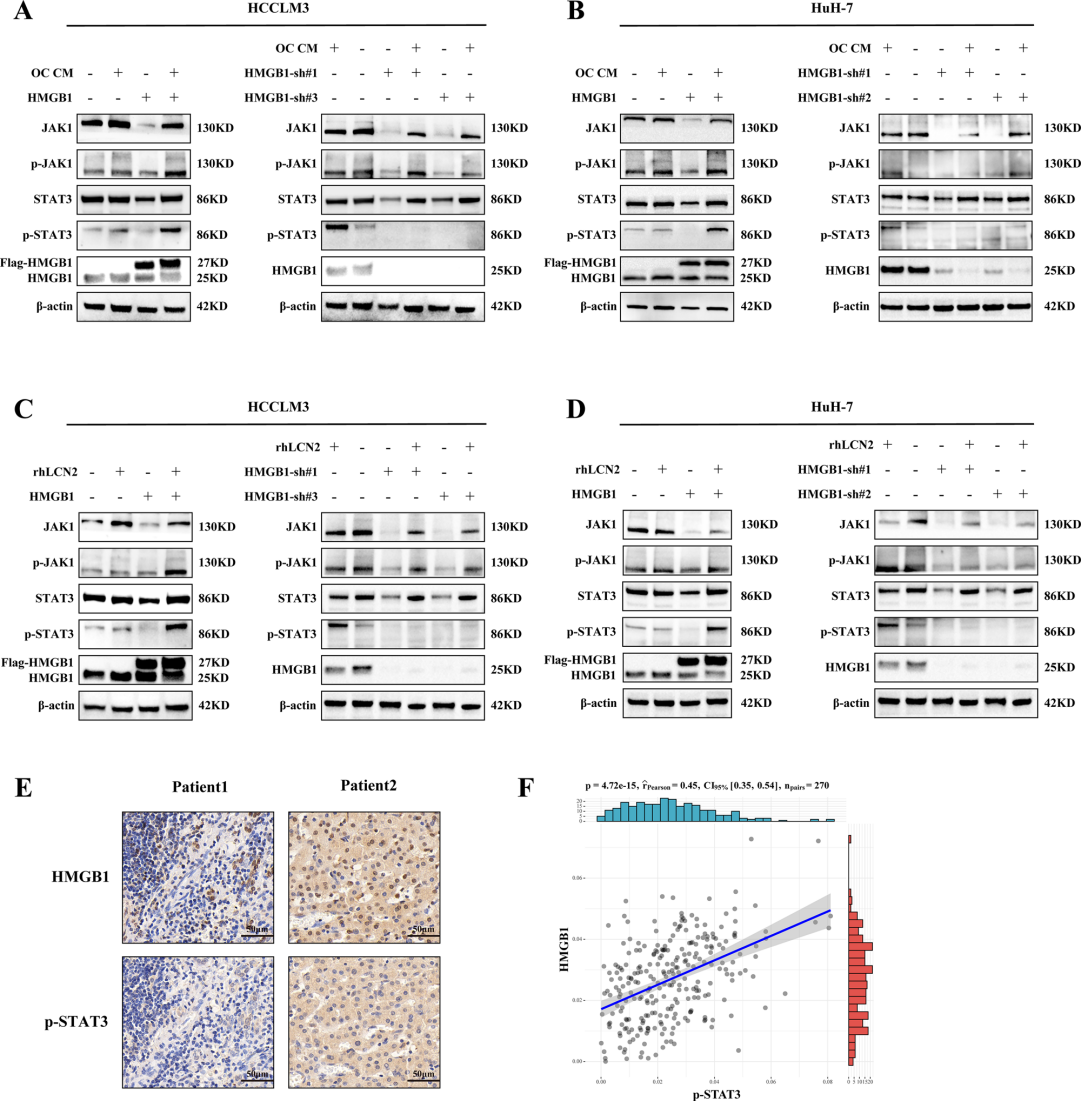
Osteoclast-derived LCN2 activates the JAK1/STAT3 signaling pathway in HMGB1-overexpressing HCC cells. Western blot analysis of JAK1, STAT3, and their phosphorylated forms (p-JAK1 and p-STAT3) in HCCLM3 (**A**) and HuH-7 (**B**) cells treated with OC CM for 24 h. JAK1/STAT3 activation was observed in HMGB1-overexpressing cells but was abolished upon HMGB1 knockdown. Western blot analysis showing that rhLCN2 treatment (75 ng/mL for 15 min) promotes JAK1 and STAT3 phosphorylation in HMGB1-overexpressing HCCLM3 (**C**) and HuH-7 (**D**) cells, while HMGB1 knockdown diminishes this effect. **E** Immunohistochemical staining of HMGB1 and p-STAT3 in clinical HCC tissues from two representative patients. where HMGB1-high samples exhibit increased STAT3 phosphorylation. **F** Scatter plot illustrating a positive correlation between HMGB1 expression and p-STAT3 levels in clinical HCC samples.

Given the established role of HMGB1 in activating the JAK1/STAT3 signaling pathway, which contributes to disease progression [26]. We explored whether osteoclast-derived LCN2 influences this pathway in HMGB1-overexpressing HCC cells. Incubation of HCCLM3 cells with OC CM significantly increased JAK1 and STAT3 phosphorylation in HMGB1-overexpressing cells, but not in controls. Knockdown of HMGB1 abolished the OC CM-induced activation of JAK1 and STAT3 (Fig. 5A). Similar results were observed in HuH-7 cells (Fig. 5B). To assess the specific contribution of LCN2, treatment with rhLCN2 similarly increased JAK1/STAT3 phosphorylation in both HCCLM3 and HuH-7 cells overexpressing HMGB1, while HMGB1 knockdown diminished this effect (Fig. 5C, D). Consistent with these findings, immunohistochemical staining of clinical HCC tissues revealed a positive correlation between HMGB1 expression and STAT3 phosphorylation (Fig. 5E, F). Together, these results demonstrate that osteoclast-derived LCN2 activates the JAK1/STAT3 signaling pathway specifically in HMGB1-overexpressing HCC cells.

### STAT3 is essential for HMGB1-mediated bone metastasis

**Fig. 6:**
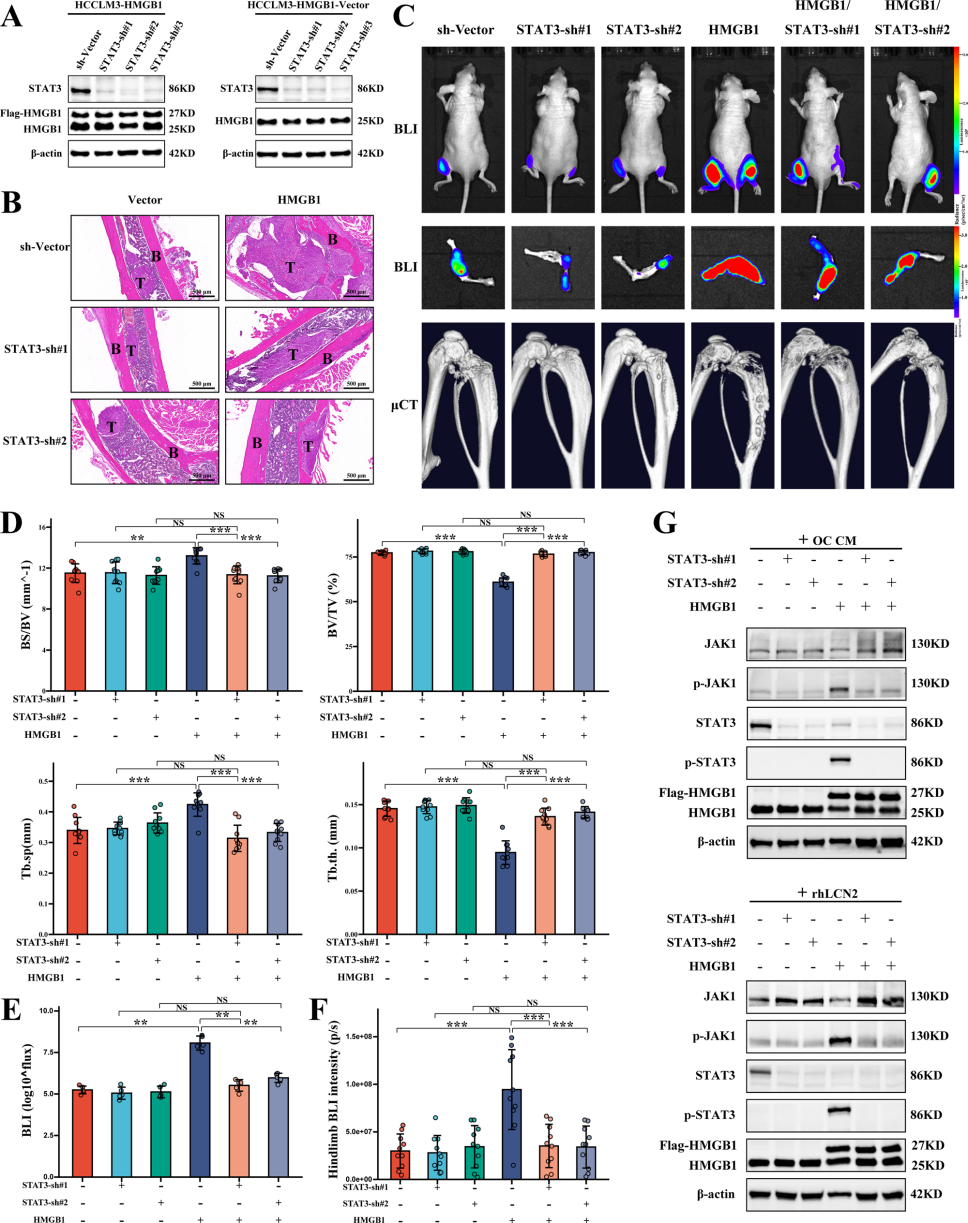
STAT3 Knockdown Inhibits HMGB1-Mediated Bone Metastasis. **A** Western blot analysis confirming effective STAT3 knockdown in control and HMGB1-overexpressing HCCLM3 cells. **B** HE staining of bone sections showing reduced tumor infiltration and osteolytic destruction at the bone-tumor interface following STAT3 knockdown. **C** BLI and μCT imaging showing that STAT3 knockdown reduces HMGB1-enhanced bone metastasis and osteolytic lesions. **D** Quantification of μCT revealing reduced osteolytic lesions in mice with STAT3-knockdown HMGB1-overexpressing HCCLM3 cells. **E** In vivo BLI quantifying reduced tumor burden in mice with STAT3-knockdown HMGB1-overexpressing cells. **F** Ex vivo BLI of hind limbs assessing decreased tumor burden in the STAT3-knockdown group. **G** Western blot analysis showing that STAT3 knockdown abolishes the phosphorylation of STAT3 induced by OC CM and rhLCN2 in HMGB1-overexpressing HCCLM3 cells.

To assess whether STAT3 functions downstream of HMGB1 in promoting bone metastasis, STAT3 was knocked down in both control and HMGB1-overexpressing HCCLM3 cells, as validated by Western blot analysis (Fig. 6A). Histological analysis further revealed that STAT3 knockdown markedly decreased tumor infiltration and osteolytic destruction at the tumor-bone interface (Fig. 6B). In mouse bone metastasis model, STAT3 knockdown significantly decreased HMGB1-enhanced tumor burden and osteolytic lesions. This was evidenced by BLI and μCT in Fig. 6C, μCT indicating decreased osteolytic lesion volume, increased BV/TV and Tb.th, and reduced BS/BV and Tb.Sp (Fig. 6D). In vivo BLI confirmed a significant tumor burden reduction (Fig. 6E), while ex vivo BLI showed decreased tumor signal intensity (Fig. 6F). Mechanistically, STAT3 knockdown inhibited the phosphorylation of STAT3 induced by OC CM and rhLCN2 in tumor cells (Fig. 6G).

To evaluate the therapeutic potential of STAT3 inhibition, mice inoculated with HMGB1-overexpressing HCCLM3 cells were treated with intraperitoneal injections of the STAT3 inhibitor Napabucasin (Napa). Napa effectively suppressed tumor growth and osteolysis, as evidenced by reduced tumor burden and SREs (Supplementary Fig. S5A, B). BLI results confirmed decreased tumor burden (Supplementary Fig. S5C, D), and Napa treatment inhibited STAT3 activation induced by OC CM and rhLCN2 in HMGB1-overexpressing cells (Supplementary Fig. S5E). These results collectively demonstrate that STAT3 plays a pivotal regulatory role in the HMGB1-LCN2 axis, contributing to tumor progression and osteolysis, and highlight its potential as a therapeutic target in HCC bone metastasis.

### HMGB1 antagonist dipotassium glycyrrhizinate inhibits bone metastasis and JAK1/STAT3 activation

To evaluate the therapeutic efficacy of targeting HMGB1 in bone metastasis, the HMGB1 antagonist Dipotassium Glycyrrhizinate (Dg) was used. Mice were intratibial injected with HCCLM3 cells overexpressing HMGB1 or Vctor controls, and Dg treatment (150 mg/kg, intraperitoneally) was initiated one week post-injection and administered every other day.

**Fig. 7:**
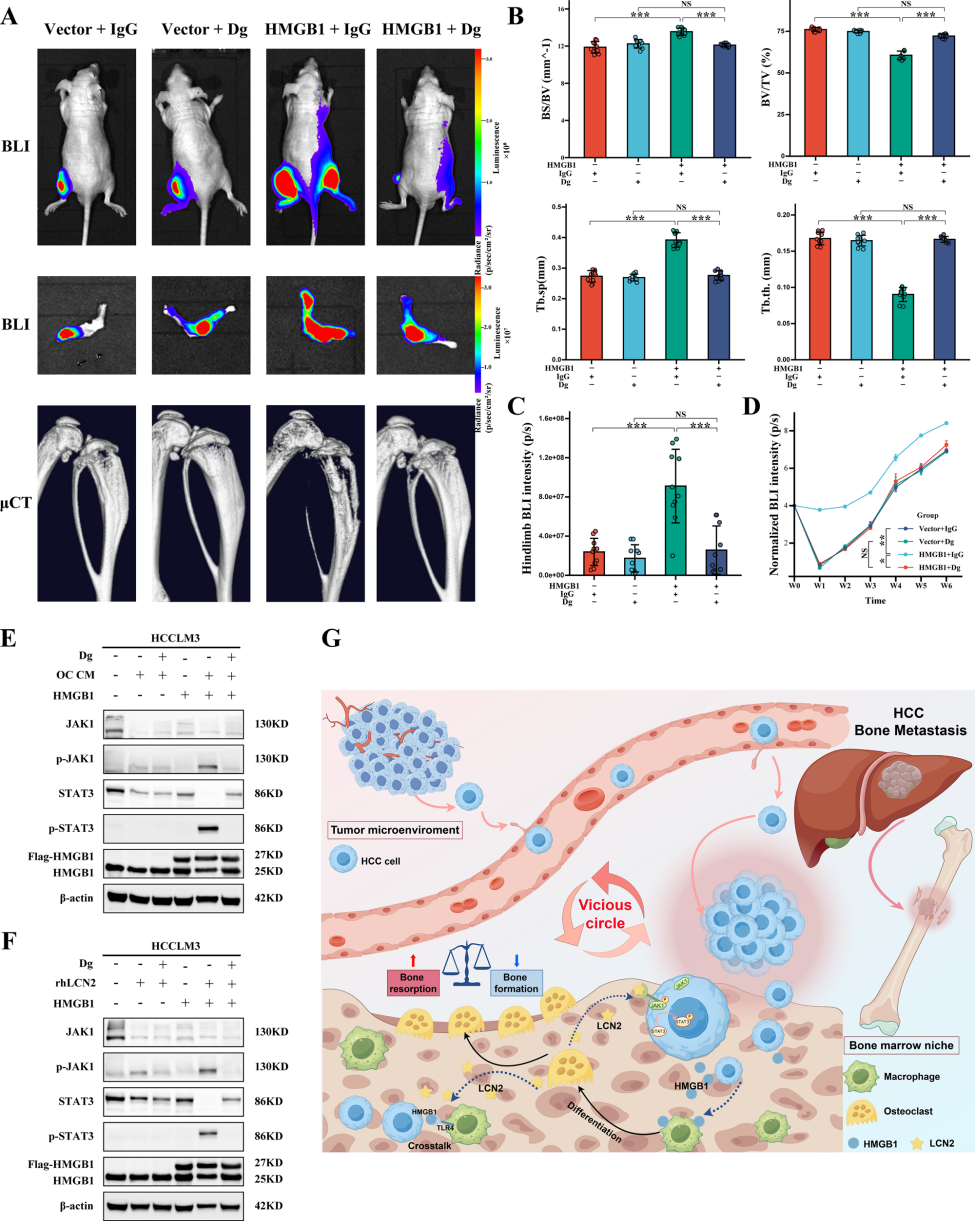
Dg inhibits HMGB1-driven bone metastasis and suppresses JAK1/STAT3 activation. **A** Representative BLI and μCT showing reduced tumor burden and inhibited bone destruction in mice injected intratibially with HMGB1-overexpressing HCCLM3 cells following Dg treatment. **B** Quantitative analysis of μCT bone parameters, demonstrating the inhibitory effects of Dg on HMGB1-driven bone destruction. **C** Ex vivo BLI quantifying a significant reduction in tumor burden with tumor signal intensity decreasing. **D** Multi-time point in vivo BLI showing a progressive reduction in tumor burden, with photon flux intensity declining. **E**, **F** Western blot analysis showing that Dg treatment (100 μM, 24 h) suppresses JAK1 and STAT3 phosphorylation in HMGB1-overexpressing HCCLM3 cells stimulated with OC CM (24 h) or rhLCN2 (75 ng/mL for 15 min). **G** Schematic model of this study. Drawn and adapted by Figdraw (https://www.figdraw.com). HMGB1 from HCC cells induces osteoclast formation from macrophages, while LCN2 from osteoclasts fuels the loop via HMGB1-TLR4 interactions and enhances HCC malignancy via HMGB1-TLR4 and JAK1-STAT3 signaling, with therapeutic implications for advanced HCC.

BLI and μCT revealed that Dg treatment significantly reduced tumor burden and SREs in mice with HMGB1-overexpressing HCCLM3 cells compared to the IgG control group (Fig. 7A). Quantitative analysis of μCT revealed that Dg markedly inhibited bone destruction, as evidenced by decreased Tb.sp and BS/BV, and increased BV/TV and Tb.th (Fig. 7B). BLI results further confirmed the reduction in tumor burden, with ex vivo BLI showing a clear decrease in metastatic spread across hind limb tissues (Fig. 7C), while multi-time point in vivo BLI depicted a consistent decline in tumor growth (Fig. 7D).

Mechanistically, Western blot analysis showed that Dg treatment effectively inhibited JAK1 and STAT3 phosphorylation induced by OC CM in HMGB1-overexpressing HCCLM3 cells (Fig. 7E). Similar inhibition of STAT3 activation was observed with rhLCN2 treatment (Fig. 7F). Together, these findings confirm that the HMGB1 antagonist Dg effectively suppresses HMGB1-driven bone metastasis by inhibiting the JAK1/STAT3 signaling pathway, highlighting its therapeutic potential in HCC bone metastasis.

Collectively, this study elucidates a novel tumor-osteoclast crosstalk mechanism within the tumor-bone microenvironment of HCC bone metastasis, introducing a novel HMGB1-LCN2-JAK1-STAT3 axis. Here, HMGB1 from metastatic HCC cells triggers a vicious cycle by driving macrophage-to-osteoclast transdifferentiation, amplifying osteolytic spread, while osteoclast-derived LCN2 fuels this loop via HMGB1-TLR4 interactions and JAK1-STAT3 activation in HCC cells, intensifying malignancy. This comprehensive experimental evidence identifies a novel therapeutic target and indicates potential applications for improving the management of advanced HCC (Fig. 7G).

## Discussion

Bone metastasis in HCC presents a critical clinical challenge, marked by severe skeletal complications and its role as a reservoir for disseminating cancer cells, fueling systemic disease progression [27]. Unlike tumor self-seeding or cross-seeding, the bone microenvironment provides a unique niche that sustains secondary metastases by enhancing tumor cell survival and proliferation [8, 27]. The hallmark of osteolytic phenotype, primarily driven by excessive osteoclast activation, exacerbates bone destruction while creating conditions that further support tumor progression [12]. However, the molecular and cellular interactions within the bone-tumor microenvironment in HCC remain poorly understood. This study addresses this gap by unveiling the central role of HMGB1 in HCC bone metastasis. Previously, HMGB1 has been implicated in HCC progression through dendritic cell-mediated CD8^+^ T cell responses [20], metastasis via the KLF7-TLR4/PTK2 signaling axis under inflammation [28], and enhanced migration and invasion through mitochondrial transfer under hypoxic conditions [21]. Our research distinctively reveals HMGB1’s novel function in orchestrating tumor-osteoclast crosstalk, identifying the HMGB1/LCN2/JAK1/STAT3 axis as a critical driver of osteolytic bone metastasis.

This study reveals a novel mechanism by which HMGB1 drives HCC bone metastasis through orchestrating a dynamic tumor-osteoclast crosstalk. Traditionally, the bone microenvironment has been regarded as a passive site of tumor colonization, where osteoclasts mediate bone resorption and release growth factors to sustain tumor progression [9]. Our findings indicate that HMGB1 contributes to osteoclast activity, leading to LCN2 secretion, which is required for JAK1/STAT3 pathway activation in HCC cells. This establishes a self-sustaining feedback loop that amplifies osteolysis and tumor dissemination, highlighting a unique dependency on LCN2 that distinguishes the HCC bone-tumor microenvironment from other cancer contexts where STAT3 activation relies on diverse mediators. The critical role of LCN2 is substantiated by its established influence on STAT3 signaling in fibrotic [29] and inflammatory microenvironments [30], providing a strong scientific basis for our mechanistic insights. The JAK1/STAT3 pathway, well-established for its oncogenic roles in cellular proliferation, immune evasion, and metastatic dissemination [11, 26], is unveiled in this study as a pivotal mediator within the osteoclast-tumor signaling axis. LCN2, previously implicated in immune modulation and metastasis in other cancers [31, 32], is identified as a key effector linking osteoclast activity to tumor cell aggressiveness in HCC. By integrating osteoclast-driven bone resorption with tumor cell signaling, HMGB1 modifies the bone microenvironment into a dynamic and active participant in metastatic progression. These findings illuminate the potential of targeting the HMGB1/LCN2/JAK1/STAT3 signaling axis as a therapeutic approach, with the capacity to disrupt the self-reinforcing pathological feedback loop that exacerbates tumor growth and osteolytic destruction in HCC bone metastasis.

Despite the widespread use of bisphosphonates and denosumab for managing bone metastases, their therapeutic efficacy remains largely restricted to mitigating SREs without effectively halting the systemic progression of metastatic cancer [33–35]. Recent evidence suggests that combination therapies targeting the crosstalk between tumor cells and the bone microenvironment may provide superior outcomes by disrupting the feedback loops that sustain tumor growth and bone resorption [36]. Previous studies have demonstrated that HMGB1-targeted approaches, including the application of Dg, can attenuate inflammation and tumor progression [37, 38], their potential in bone metastasis has remained unexplored until now. Additionally, STAT3 inhibitors like Napa have exhibited promising results in dismantling tumor-driven immunosuppressive environments and amplifying chemotherapy efficacy [39–41]. Our study integrates these insights, proposing that targeting the HMGB1/LCN2/JAK1/STAT3 axis can effectively disrupt tumor-osteoclast crosstalk, suppress osteolytic bone resorption, and potentially curtail systemic tumor dissemination. This therapeutic approach not only holds promise for improving skeletal outcomes but also offers a systemic benefit by addressing the molecular drivers of metastasis. In this study, Dg significantly reduced osteoclast activation and tumor burden in vivo, offering a potential upstream intervention to disrupt the tumor-osteoclast interaction. By blocking HMGB1 release, Dg prevents the initiation of the molecular cascade that sustains the osteolytic microenvironment. Additionally, Napa effectively inhibited JAK1/STAT3 signaling in HMGB1-overexpressing tumor cells exposed to OC-CM or rhLCN2. Future investigations into the synergistic potential of combining HMGB1 and STAT3 inhibitors with current anti-osteoclast therapies could offer a transformative strategy for managing HCC bone metastasis, reducing both skeletal complications and systemic tumor progression.

This study advances the understanding of tumor-osteoclast crosstalk in HCC bone metastasis, yet several dimensions warrant further exploration to refine these insights. The upstream regulation of HMGB1 secretion—whether governed by intrinsic tumor factors, microenvironmental signals, or systemic influences—remains an unresolved question necessitating detailed investigation. Moreover, the applicability of this axis beyond HCC, particularly in osteolytic metastases such as those in breast or prostate cancer, requires comparative studies to assess its broader relevance. Furthermore, the prognostic value of serum HMGB1, as suggested by its association with adverse outcomes in advanced HCC post-treatment [42], merits further study as a non-invasive biomarker. Future research will conduct inhibition experiments, such as using RANKL inhibitors or osteoclast-specific genetic knockdowns, to elucidate the causal interactions between HMGB1-driven osteoclast activation and HCC bone metastasis, thereby refining the theoretical framework of the axis. Additionally, preclinical studies employing nanotechnology-based delivery systems could enhance therapeutic precision within the bone-tumor microenvironment, reducing systemic effects and improving clinical outcomes. These efforts promise to deepen the understanding and management of HCC bone metastasis.

In conclusion, this study establishes a critical molecular framework by identifying the HMGB1/LCN2/JAK1/STAT3 axis as a central mechanism in the development of osteolytic bone metastasis in HCC. Our comprehensive analysis shows that HMGB1 orchestrates osteoclast activation, induces LCN2 signaling, and activates the JAK1/STAT3 pathway, creating a feedback loop that enhances bone destruction and tumor dissemination within the bone-tumor microenvironment. This finding highlights a promising therapeutic target and contributes to the understanding of HCC metastasis, providing a critical step toward translating molecular discoveries into clinically effective strategies for managing bone metastasis in HCC.

## Supplementary information

Supplementary Figure legend

Supplementary Materials and Methods

Full uncropped Gels and Blots

Figure S1

Figure S2

Figure S3

Figure S4

Figure S5

Table S1

Table S2

## Data Availability

Bulk RNA-seq data generated in this study have been deposited in the NCBI’s Sequence Read Archive (SRA) under BioProject accession number PRJNA1208261 (https://dataview.ncbi.nlm.nih.gov/object/PRJNA1208261?reviewer=brqfoq1nq6d4uhqlden4o6ane7). Publicly available scRNA-seq datasets included in the analysis are from the GEO database, all data are accessible through their respective GEO entries.
