# Supplementary material for: HMGB1 orchestrates tumor-osteoclast crosstalk to drive bone metastasis in hepatocellular carcinoma
Source: Cell Death Dis. 2025 Oct 7;16(1):712. doi: 10.1038/s41419-025-08037-6 (PMC12504543; doi:10.1038/s41419-025-08037-6)
Supplement: Supplementary file 1 — Supplementary Figure legend [file 41419_2025_8037_MOESM1_ESM.docx]

**Figure S1. Gene Set Enrichment Analysis in HCC Bone Metastasis Models. (A)** GSEA plot showing enrichment of the "GOBP_lymphocyte activation involved in immune response" pathway in the normal group, indicating enhanced anti-tumor immune activity. **(B)** GSEA plot illustrating enrichment of the "HALLMARK_epithelial mesenchymal transition" pathway in the Vector group, suggesting enhanced metastatic potential. **(C)** GSEA plot highlighting enrichment of the "MP_increased metastatic potential" pathway in the HMGB1 group, supporting its pro-metastatic role. **(D)** GSEA plot demonstrating enrichment of the "HALLMARK_epithelial mesenchymal transition" pathway in the HMGB1 group, reinforcing metastatic characteristics. **(E)** GSEA plot indicating enrichment of "GOBP_bone_resorption" and "GOBP_bone_remodeling" pathways in the HMGB1 group, consistent with increased osteoclast activity.

**Figure S2. HMGB1 Knockdown Attenuates HCC Bone Metastasis and Skeletal Complications In Vivo. (A)** Western blot analysis validating HMGB1 knockdown in HuH-7 and HCCLM3 cell lines. **(B)** Representative images from BLI and μCT demonstrating reduced bone metastasis and osteolytic lesions in mice injected with HMGB1-knockdown cells. **(C)** Quantification of μCT bone parameters, confirming reduced skeletal destruction with decreased Tb.sp and BS/BV, and increased BV/TV and Tb.th in the HMGB1-knockdown group. **(D)** In vivo BLI signal curve over time, indicating suppressed bone metastasis progression in the HMGB1-knockdown group. **(E)** Ex vivo BLI signal at the experimental endpoint, confirming reduced tumor burden in the HMGB1-knockdown group. **(F)** Histological images (HE and TRAP) illustrating reduced osteolytic bone destruction and TRAP-positive osteoclast areas following HMGB1 knockdown. **(G)** Quantitative analysis of TRAP-positive osteoclast areas.

**Figure S3. GSEA of HMGB1-Induced Osteoclast and Myeloid Cell Pathways. (A)** GSEA plot illustrating significant enrichment of the "GOBP_osteoclast_differentiation" gene set in the rhHMGB1-treated group. **(B)** GSEA plot demonstrating significant enrichment of the "BROWN_myeloid_cell_development_up" gene set in the rhHMGB1-treated group. **(C)** GSEA plot showing enrichment of the "BROWN_myeloid_cell_development_dn" gene set in the control group.

**Figure S4. LCN2 Enhance HMGB1-TLR4 Interaction in the Tumor-Bone Microenvironment. (A)** Immunofluorescence staining images of RAW264.7 cells treated with rhHMGB1 at varying concentrations, demonstrating increased TLR4 expression. **(B)** Immunofluorescence analysis images showing a dose-dependent increase in TLR4 expression in RAW264.7 cells treated with rhLCN2. **(C)** Co-IP images confirming the interaction between HMGB1 and TLR4, with enhanced binding observed with increasing concentrations of rhLCN2. **(D)** Quantification of Co-IP results, illustrating the concentration-dependent enhancement of the HMGB1-TLR4 interaction by rhLCN2.

**Figure S5. Therapeutic Inhibition of STAT3 by Napa Suppresses HMGB1-Driven Bone Metastasis. (A)** Representative images from BLI and μCT analysis showing that Napa treatment suppresses HMGB1-driven bone metastasis and osteolysis in vivo. **(B)** Quantification of μCT revealing reduced osteolytic lesions in mice model with Napa. **(C)** In vivo BLI quantifying reduced tumor burden in mice with Napa. **(D)** Ex vivo BLI of hind limbs at the experimental endpoint, assessing decreased tumor burden in the Napa group**. (E)** Western blot analysis confirming that Napa (5 μM, 24 hours) inhibits STAT3 phosphorylation induced by OC CM and rhLCN2 in HMGB1-overexpressing HCCLM3 cells.
