# Supplementary material for: HMGB1 orchestrates tumor-osteoclast crosstalk to drive bone metastasis in hepatocellular carcinoma
Source: Cell Death Dis. 2025 Oct 7;16(1):712. doi: 10.1038/s41419-025-08037-6 (PMC12504543; doi:10.1038/s41419-025-08037-6)
Supplement: Supplementary file 2 — Supplementary Materials and Methods [file 41419_2025_8037_MOESM2_ESM.docx]

**Materials and Methods**

**Cell lines**

The human hepatocellular carcinoma (HCC) cell lines HCCLM3 and HuH-7, the murine macrophage cell line RAW264.7, and the human embryonic kidney 293T cells were obtained from the Cell Bank of the Chinese Academy of Sciences (Shanghai, China; https://www.cellbank.org.cn). All cell lines were verified to be mycoplasma-free. Cells were cultured in DMEM (Gibco, USA) supplemented with 10% FBS (Biological Industries, Israel), 1% GlutaMAX (Gibco, USA), 1% sodium pyruvate (Gibco, USA), and 1% penicillin-streptomycin-gentamicin (Beyotime, China) and maintained at 37℃ in a humidified incubator with 5% CO₂.

**Lentivirus construction and cell transfection**

Lentiviral vectors pcSLenti-EF1-EGFP-P2A-Puro-CMV-HMGB1-3×Flag-WPRE and pcSLenti-EF1-EGFP-P2A-Puro-CMV-MCS-3×Flag-WPRE were purchased from OBiO Technology (Shanghai, China), and hU6-MCS-CBh-gcGFP-IRES-puromycin-HMGB1-RNAi and hU6-MCS-Ubiquitin-mCherry-IRES-Neomycin-STAT3-RNAi from Shanghai Genechem Ltd. The short hairpin RNA (shRNA) sequences targeting HMGB1 and STAT3 are provided in Supplementary **Table S1**. Stable HCC cell lines were established by infecting cells with lentiviruses encoding HMGB1 or shRNA targeting HMGB1 or STAT3 (constructs: HMGB1-sh#1, HMGB1-sh#2, HMGB1-sh#3, STAT3-sh#1, STAT3-sh#2, STAT3-sh#3)) in the presence of 4 μg/mL polybrene. Infected cells were selected using 5 μg/mL puromycin and/or 700 μg/mL G418 (Geneticin). Stable transfection was confirmed via Western blot.

**Antibodies and reagents**

The antibodies used for Western blotting, immunoprecipitation, immunofluorescence, and immunohistochemistry included: β-actin (Beyotime, AF5003), HMGB1 (Abcam, ab79823), TLR4 (Proteintech, 19811-1-AP), HMGB1 for immunoprecipitation (Santa Cruz Biotechnology, HAP46.5), GPC3 (ABclonal, A11686), GFP (Abcam, ab183734), F4/80 (Abcam, ab300421), STAT3 (CST, 4904S), phosphorylated STAT3 (p-STAT3) (CST, 9145S), JAK1 (CST, 3344S), and p-JAK1 (CST, 3331S). Recombinant proteins included: human HMGB1 (MedChemExpress, HY-P70570), human LCN2 (MedChemExpress, HY-P71156), recombinant mouse M-CSF protein (R&D Systems, 416-ML-010), and recombinant mouse RANKL (TRANCE/TNFSF11) (R&D Systems, 462-TEC-010). The inhibitors used were: Napabucasin (MedChemExpress, HY-13919) and Dipotassium glycyrrhizinate (MedChemExpress, HY-N0184A). ELISA kits included: Mouse Neutrophil gelatinase-associated lipocalin (NGAL)(LCN2) (ABclonal, RK03063), and HMGB1 (ABclonal, RK06737).

**Osteoclastogenesis assays**

RAW264.7 cells (1 × 10⁵) were seeded onto 24-well plates containing glass coverslips (Biosharp, China) and cultured in conditioned medium (CM) derived from HCC cells, mixed at a 1:3 ratio with α-MEM supplemented with 10% FBS, 20 ng/mL macrophage colony-stimulating factor (M-CSF), and 50 ng/mL RANKL. Media were refreshed every two days, and osteoclast differentiation was assessed on day 6. Cells cultured on plastic dishes were fixed with 4% paraformaldehyde and stained for tartrate-resistant acid phosphatase (TRAP) using commercial kit (Solarbio, G1492). Osteoclasts were identified as multinucleated TRAP-positive cells containing at least three nuclei, and their abundance was quantified as the number of multinucleated cells per unit length.

For the preparation of osteoclast-derived CM (OC CM), RAW264.7 cells were cultured in α-MEM supplemented with 10% FBS, 20 ng/mL M-CSF, 50 ng/mL RANKL, and 100 ng/mL rhHMGB1 for 5–7 days. Differentiated osteoclasts were washed with PBS and incubated in RANKL-free α-MEM containing 1% FBS for 24 hours. The resulting supernatant was filtered and collected for downstream applications.

**Bone resorption pit assay**

To evaluate the bone-resorbing activity of osteoclasts, RAW264.7 cells (1 × 10⁵) were seeded onto bone slices (IDS, DT-1BON1000-96) and cultured for 9 days in CM derived from HCC cells. Media were replenished every two days. After 9 days, the bone slices were fixed with 2.5% glutaraldehyde, and adherent cells were removed by sonication. Resorption lacunae were visualized using scanning electron microscopy (SEM), and three random fields per bone slice were selected for analysis. The resorption pit areas were quantified using Image-Pro Plus software (Media Cybernetics).

**Bulk RNA sequencing**

RNA-seq was conducted on mouse bone marrow samples and RAW264.7-derived osteoclasts. Total RNA was extracted using TRIzol reagent (Invitrogen, 10296010), and its quality was assessed with a NanoDrop spectrophotometer and an Agilent 2100 Bioanalyzer. RNA libraries were prepared using the VAHTS Universal V6 RNA-seq Library Prep Kit and sequenced on an Illumina NovaSeq 6000 platform (OE Biotech Co., Ltd., Shanghai, China) to generate 150 bp paired-end reads. Reads were quality-filtered using fastp and aligned to the mouse reference genome (mm10) with HISAT2. Differential gene expression was analyzed using Limma-voom[1], with genes meeting |log2 fold change| > 1 and FDR < 0.05 considered significant. Gene Set Enrichment Analysis (GSEA) was performed using GSEA software v4.3.3 (<http://software.broadinstitute.org/gsea/>) to evaluate enrichment of bone resorption and tumor progression pathways, with significance thresholds of *P* < 0.05 and FDR < 0.25.

**Single-cell RNA sequencing**

The scRNA-seq analysis was conducted using the publicly available GSE149614 dataset[2], which includes 21 samples from 10 HCC patients. Samples were derived from primary tumors (PT), non-tumor liver tissues (NTL), portal vein tumor thrombi (PVTT), and metastatic lymph nodes (MLN). Data processing followed established workflows outlined in our previous publication[3]. The Seurat package was employed for data processing and clustering[4], while Harmony was used for batch effect correction[5]. Cell annotations were conducted using manual marker-based methods and automated tools, such as singleR[6] and sctype[7] , to ensure unbiased identification. Malignant and non-malignant epithelial cells were differentiated through the Copykat algorithm by inferring copy number variations[8].

**Cell migration assays**

The migratory ability of HCC cells was evaluated using wound healing assay. HCC cells (8 × 10⁵/mL) were seeded into 6-well plates. After cell adherence, a wound was created by dragging a 200-μL pipette tip across the cell monolayer. Cells were incubated in CM derived from osteoclasts mixed with serum-free medium or DMEM containing 75 ng/mL recombinant LCN2 protein without FBS for 48 hours. Wound closure was visualized using an inverted microscope (ZEISS Axio Vert.A1) and quantified using ImageJ software to measure the migration distance of cells.

**Cell invasion assays**

The invasive capacity of HCC cells was assessed using transwell chambers with 8-μm pore inserts pre-coated with Matrigel (Corning, USA), following the manufacturer’s protocol. Briefly, 500 μL of CM derived from osteoclasts with 10% FBS or DMEM containing 10% FBS and 75 ng/mL recombinant LCN2 protein was added to the lower chamber. HCC cells (1 × 10⁵) in 200 μL of FBS-free DMEM were seeded into the upper chamber. After 48 hours at 37°C, non-invasive cells on the upper surface of the membrane were removed with a cotton swab. Invasive cells on the underside were fixed with 4% paraformaldehyde, stained with crystal violet (Beyotime, China), and counted in random fields under a microscope. All experiments were performed in triplicate.

**Western blotting**

Proteins were extracted using RIPA buffer (Beyotime, P0013B) with protease and phosphatase inhibitors (Beyotime, China), denatured in 5× SDS loading buffer (Beyotime, China) and denatured in 5× SDS loading buffer and transferred onto polyvinylidene difluoride (PVDF) membranes. blocking was performed with rapid blocking buffer (Epizyme, China) for 15 minutes at room temperature. Membranes were incubated with primary antibodies overnight at 4°C, followed by HRP-conjugated secondary antibodies for 1 hour at room temperature. Protein bands were visualized using enhanced chemiluminescence (ECL).

**Quantitative real-time polymerase chain reaction (qRT-PCR)**

Total RNA was extracted using Trizol reagent (Invitrogen, USA) according to the manufacturer’s protocol, RNA concentration and purity were assessed using a NanoDrop spectrophotometer (Thermo, USA). One microgram of RNA was reverse transcribed into complementary DNA (cDNA) using the PrimeScript™ RT reagent kit (Takara Bio, Japan). qRT-PCR was performed using SYBR qPCR Master Mix (Yeasen, China) on an ABI Q6 PCR system. Gene expression levels were quantified using the 2^⁻ΔΔCT^ method, with GAPDH as the endogenous control. Primer sequences are listed in Supplementary Table S2.

**Co-immunoprecipitation (Co-IP)**

Total proteins were extracted using IP lysis buffer (Beyotime, China) supplemented with protease and phosphatase inhibitors (Beyotime, China). Lysates were centrifuged at 12,000 rpm for 15 minutes at 4°C, and the supernatants were collected. For immunoprecipitation, the supernatants were pre-cleared with mouse IgG (Proteintech, B900620) overnight at 4°C or incubated with specific IP antibodies for 2 hours at 4°C, followed by the addition of Protein A/G Plus-Agarose beads (Santa Cruz Biotechnology, sc-2003) and incubation overnight at 4°C. The beads were washed three times with pre-cooled IP buffer and resuspended in 2× SDS loading buffer (Beyotime, China). Protein-protein interactions were analyzed via western blotting. For quantification of Co-IP results, densitometric analysis of Western blot bands was performed using ImageJ software. The binding activity was calculated as the ratio of normalized intensities of TLR4 co-precipitated with HMGB1. Statistical analysis was conducted using one-way ANOVA with Tukey’s post-hoc test to compare ratios across rhLCN2 concentrations

**Mouse models**

BALB/c nude mice (5 weeks old, male) were purchased from Shanghai South Model Biotechnology Co., Ltd. and used for xenograft experiments, with randomly allocated to experimental groups prior to treatment initiation. A mice model of bone metastasis from HCC was established by intratibial injection of HCCLM3 cells (1×10⁶ cells/mL). Dipotassium glycyrrhizinate (Dg) treatment commenced one week post-tumor inoculation via intraperitoneal injection (150 mg/kg every other day), while Napabucasin (40 mg/kg) was administered intraperitoneally every two days.

Bone metastasis progression was monitored weekly through bioluminescence imaging (BLI) using the IVIS Spectrum CT system (PerkinElmer) after D-luciferin (75 mg/kg) injection. At week 6, osteolytic lesions were assessed via micro-computed tomography (Quantum GX2, PerkinElmer), and single-photon emission computed tomography (SPECT) data were acquired using the VivoQuant system (MOLECUBES, Belgium).

**In vivo quantification of osteoclast number**

Hind limbs were fixed in 4% paraformaldehyde, decalcified in 14.3% EDTA for 4 days at 37°C with daily EDTA changes, and embedded in paraffin. Sections were stained with Mayer’s hematoxylin solution for hematoxylin and eosin (HE) staining or with a tartrate-resistant acid phosphatase (TRAP) staining kit (Solarbio, G1492) following the manufacturer’s instructions. Osteoclast numbers were quantified as multinucleated TRAP-positive cells per unit length.

**Enzyme-linked immunosorbent assay (ELISA)**

The concentrations of HMGB1 and LCN2 (NGAL) levels in the culture medium were quantified using the HMGB1 (ABclonal, RK06737) and NGAL (ABclonal, RK03063) ELISA Kit following the manufacturer’s protocols. Absorbance was measured at 450 nm using a spectrophotometer.

**Multiplex immunohistochemical (mIHC)**

Tissue slides were deparaffinized with xylene (twice for 15 minutes each) and rehydrated through a graded ethanol series (100%, 95%, 85%, and 75%, 5 minutes each). Antigen retrieval was performed using a microwave method with 10 mM citrate buffer (pH 6.0). Endogenous peroxidase activity was blocked with 3% H₂O₂ for 15 minutes. To reduce nonspecific binding, sections were incubated in 10% normal goat serum (Beyotime, C0265) for 30 minutes at room temperature. Primary antibodies against HMGB1 (1:200, Abcam, ab79823), GFP (1:500, Abcam, ab183734), F4/80 (1:100, Abcam, ab300421), TLR4 (1:100, Proteintech, 19811-1-AP) and GPC3 (1:100, ABclonal, A12383) were applied overnight at 4°C. Slides were then incubated with corresponding secondary antibodies, and signal amplification was performed using a IHC kit (AiFang Biological, AFIHC025, AFIHC033) following the manufacturer’s instructions. Nuclei were counterstained with DAPI for 20 minutes. Stained slides were scanned using a digital scanner (3DHISTECH-Pannoramic MIDI, 3DHISTECH Ltd., Budapest).

For quantitative co-localization analysis of mIHC images, fluorescence intensities were measured along linear regions of interest (ROIs) in overlay images using ImageJ software. ROIs were selected to traverse areas of potential co-localization between HMGB1, GFP (HCC cells), and F4/80 (macrophages). Intensity profiles were plotted, and Pearson’s correlation coefficients were calculated using the Coloc 2 plugin to quantify co-localization between HMGB1 and other markers.

**Patients and samples**

HCC tissue samples were collected from patients undergoing surgery at Shanghai East Hospital, Tongji University, China, between January 2018 and December 2022. Inclusion criteria required a confirmed diagnosis of HCC through imaging and pathological assessment, as well as the availability of comprehensive follow-up data. Patients with a history of prior local or systemic treatment were excluded. All specimens were collected with informed consent and in compliance with protocols approved by the Ethics Committee of Shanghai East Hospital (No.2022250).

**Immunohistochemistry (IHC)**

Tissue samples were fixed in 4% paraformaldehyde (Beyotime) and embedded in paraffin. Sections were deparaffinized, rehydrated, and subjected to antigen retrieval. Blocking was performed with 5% BSA for 30 minutes at room temperature, followed by overnight incubation at 4°C with primary antibodies against HMGB1 (1:200, Abcam, ab79823) or phosphorylated STAT3 (p-STAT3) (1:200, CST, 9145S). Sections were incubated overnight at 4°C. After washing, the sections were incubated with HRP-conjugated secondary antibodies for 1 hour at room temperature. Antigen detection was carried out using a diaminobenzidine (DAB) chromogenic substrate. Staining intensities were quantified using QuPath software to ensure accurate analysis.

**Statistical analysis**

All statistical analyses were performed using R (v4.2.0). Data are presented as mean ± standard error of the mean (SEM) for continuous variables or as frequencies and percentages for categorical variables. Comparisons between two groups were conducted using the unpaired two-tailed Student’s t-test for normally distributed data or the Mann–Whitney U test for non-normally distributed data. Categorical variables were compared using the chi-square test or Fisher’s exact test when appropriate. For comparisons involving three or more groups, one-way analysis of variance (ANOVA) followed by Tukey’s post hoc test was applied. Survival analyses were performed using the Kaplan–Meier method, with significance assessed by the log-rank test. All experiments were repeated at least three times, and a *P* value < 0.05 was considered statistically significant.

**REFERENCES**

1. Law CW, Chen Y, Shi W, Smyth GK. Voom: precision weights unlock linear model analysis tools for RNA-seq read counts. Genome Biol. 2014;15:R29.

2. Lu Y, Yang A, Quan C, Pan Y, Zhang H, Li Y, et al. A single-cell atlas of the multicellular ecosystem of primary and metastatic hepatocellular carcinoma. Nat Commun. 2022;13:4594.

3. Chen Y, Meng Z, Xiang Z. HMGB2 drives tumor progression and shapes the immunosuppressive microenvironment in hepatocellular carcinoma: insights from multi-omics analysis. Front Immunol. 2024;15:1415435.

4. Hao Y, Hao S, Andersen-Nissen E, Mauck WM, Zheng S, Butler A, et al. Integrated analysis of multimodal single-cell data. Cell. 2021;184:3573-87.

5. Castanza AS, Recla JM, Eby D, Thorvaldsdóttir H, Bult CJ, Mesirov JP. Extending support for mouse data in the molecular signatures database (MSigDB). Nat Methods. 2023;20:1619-20.

6. Aran D, Looney AP, Liu L, Wu E, Fong V, Hsu A, et al. Reference-based analysis of lung single-cell sequencing reveals a transitional profibrotic macrophage. Nat Immunol. 2019;20:163-72.

7. Ianevski A, Giri AK, Aittokallio T. Fully-automated and ultra-fast cell-type identification using specific marker combinations from single-cell transcriptomic data. Nat Commun. 2022;13:1246.

8. Gao R, Bai S, Henderson YC, Lin Y, Schalck A, Yan Y, et al. Delineating copy number and clonal substructure in human tumors from single-cell transcriptomes. Nat Biotechnol. 2021;39:599-608.
