# Supplementary material for: HMGB1 orchestrates tumor-osteoclast crosstalk to drive bone metastasis in hepatocellular carcinoma
Source: Cell Death Dis. 2025 Oct 7;16(1):712. doi: 10.1038/s41419-025-08037-6 (PMC12504543; doi:10.1038/s41419-025-08037-6)
Supplement: Supplementary file 3 — Full uncropped Gels and Blots [file 41419_2025_8037_MOESM3_ESM.pdf]

Fig2A

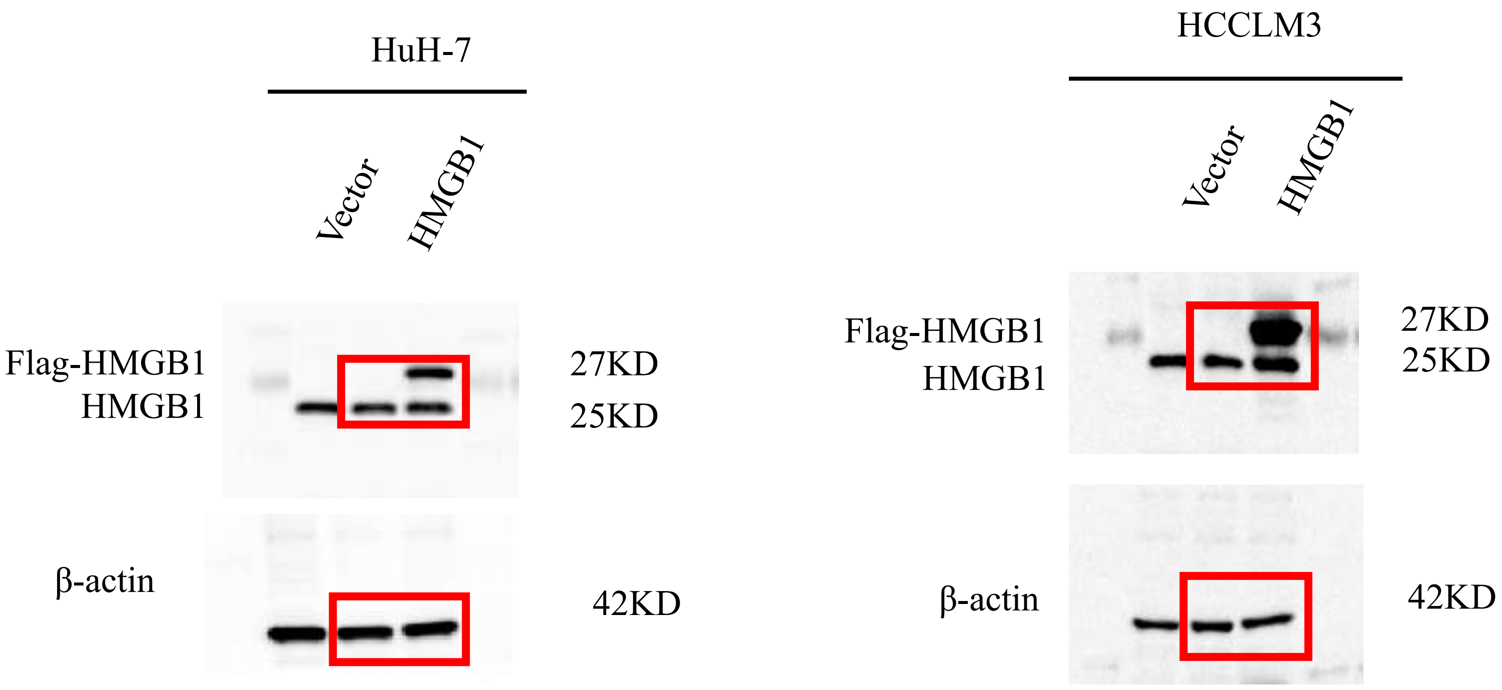
HuH-7
Vector
HMGB1
Flag-HMGB1
HMGB1
27KD
25KD
β-actin
42KD
HCCLM3
Vector
HMGB1
Flag-HMGB1
HMGB1
27KD
25KD
β-actin
42KD

FigS2A

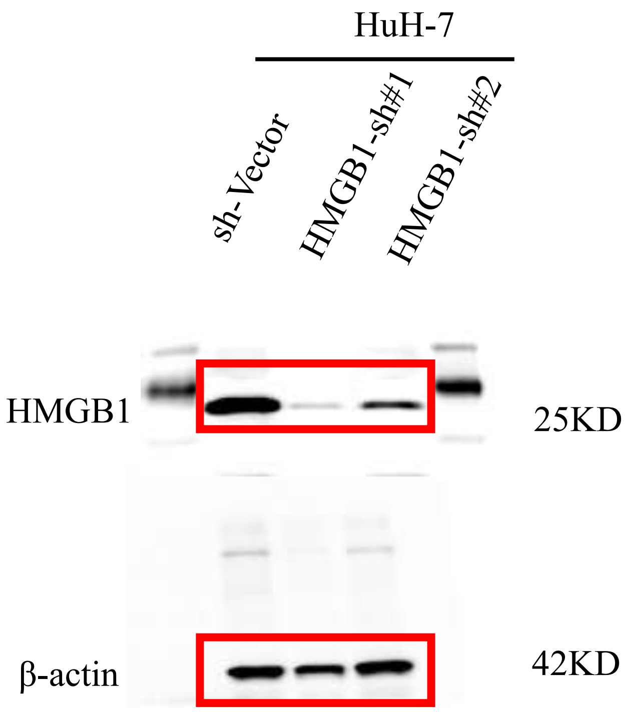
HuH-7
sh-Vector
HMGB1-sh#1
HMGB1-sh#2
HMGB1
25KD
β-actin
42KD

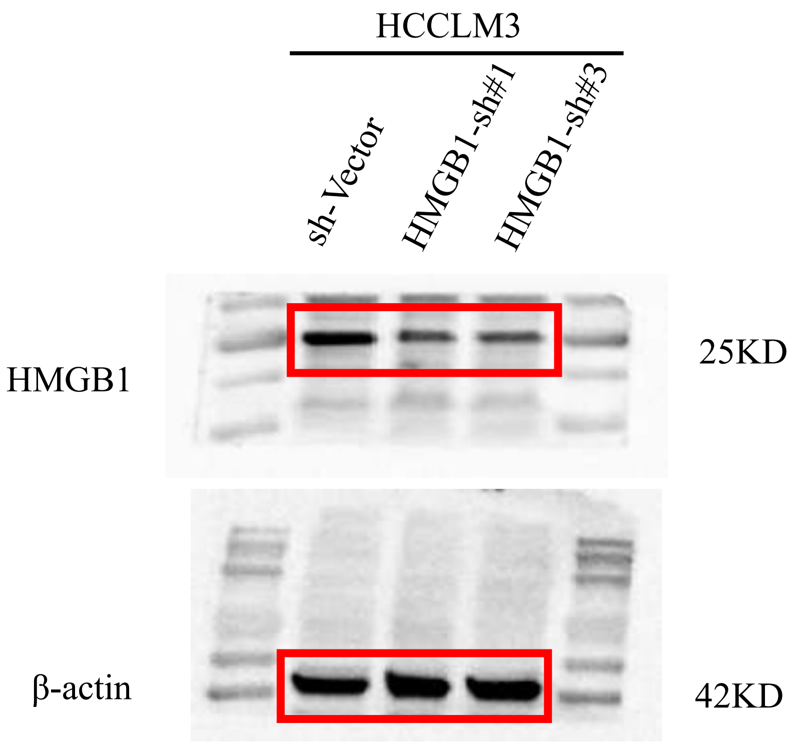
HCCLM3
sh-Vector
HMGB1-sh#1
HMGB1-sh#3
HMGB1
25KD
β-actin
42KD

FigS4C

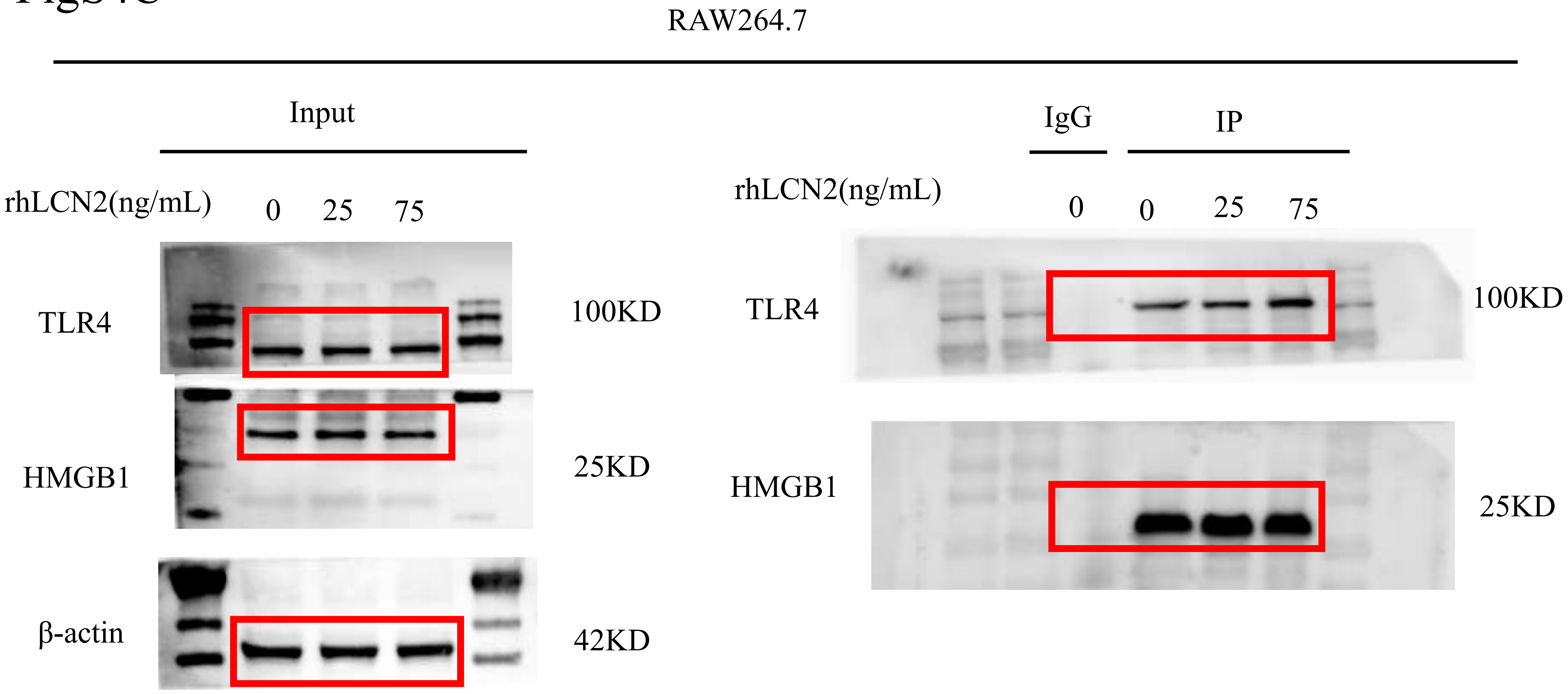
RAW264.7
Input
IgG
IP
rhLCN2(ng/mL)
0
25
75
rhLCN2(ng/mL)
0
0
25
75
TLR4
100KD
HMGB1
25KD
β-actin
42KD
TLR4
100KD
HMGB1
25KD

Fig5A

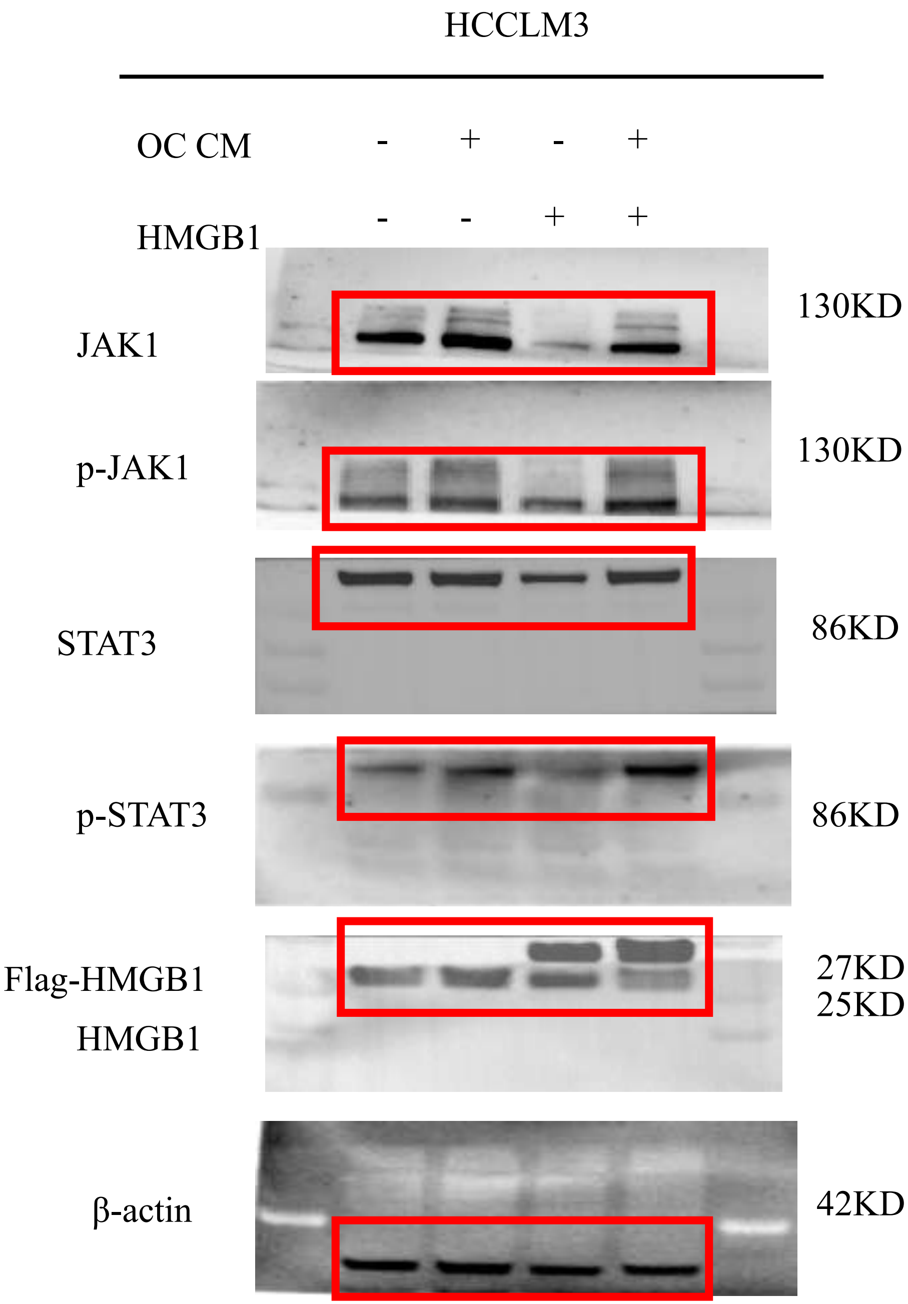
HCCLM3
OC CM - + - +
HMGB1 - - + +
JAK1
130KD
p-JAK1
130KD
STAT3
86KD
p-STAT3
86KD
Flag-HMGB1
HMGB1
27KD
25KD
β-actin
42KD

Fig5A

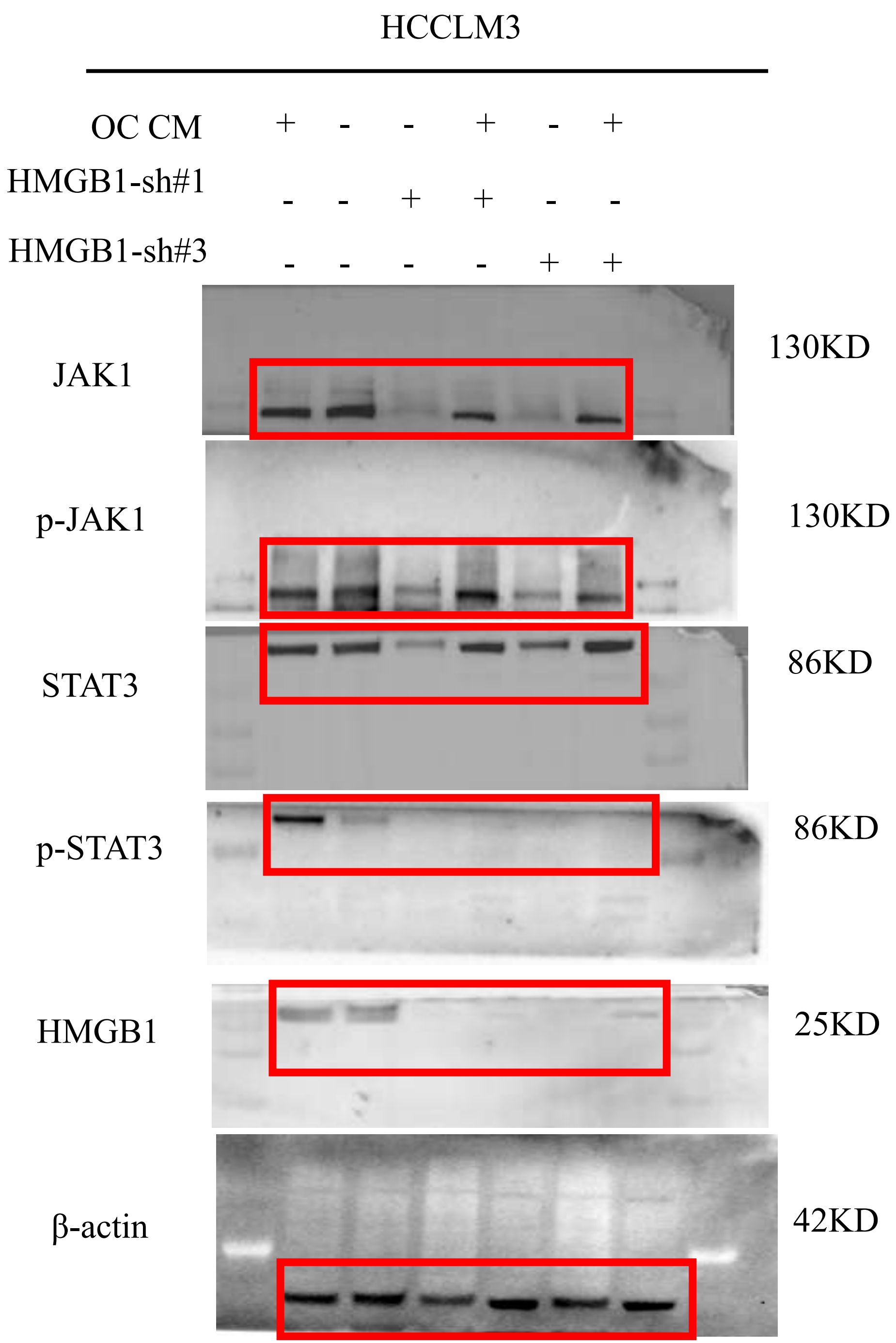

HCCLM3
OC CM + - - + - +
HMGB1-sh#1 - - + + - -
HMGB1-sh#3 - - - - + +
JAK1
130KD
p-JAK1
130KD
STAT3
86KD
p-STAT3
86KD
HMGB1
25KD
β-actin
42KD

Fig5B

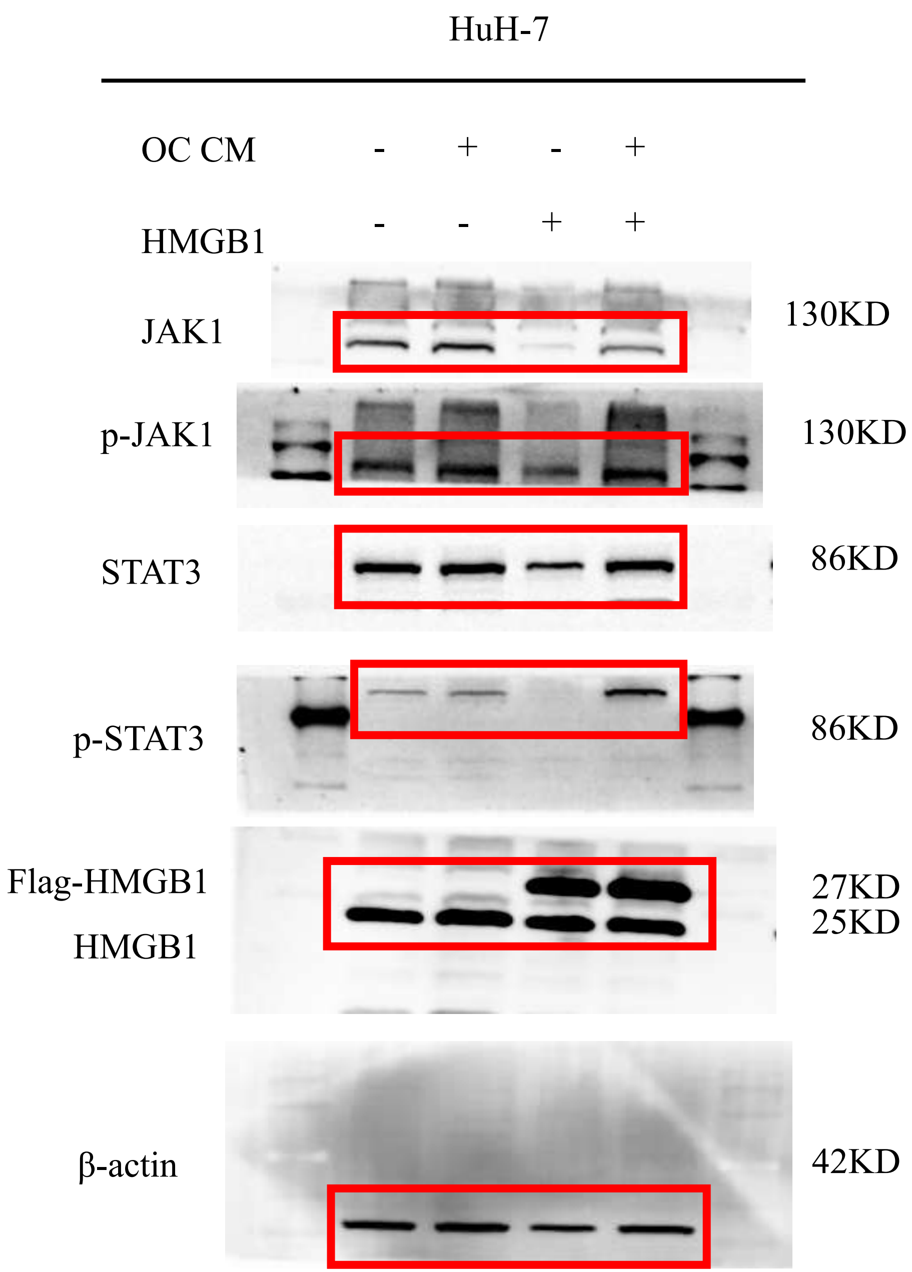
HuH-7
OC CM - + - +
HMGB1 - - + +
JAK1
130KD
p-JAK1
130KD
STAT3
86KD
p-STAT3
86KD
Flag-HMGB1
HMGB1
27KD
25KD
β-actin
42KD

Fig5B

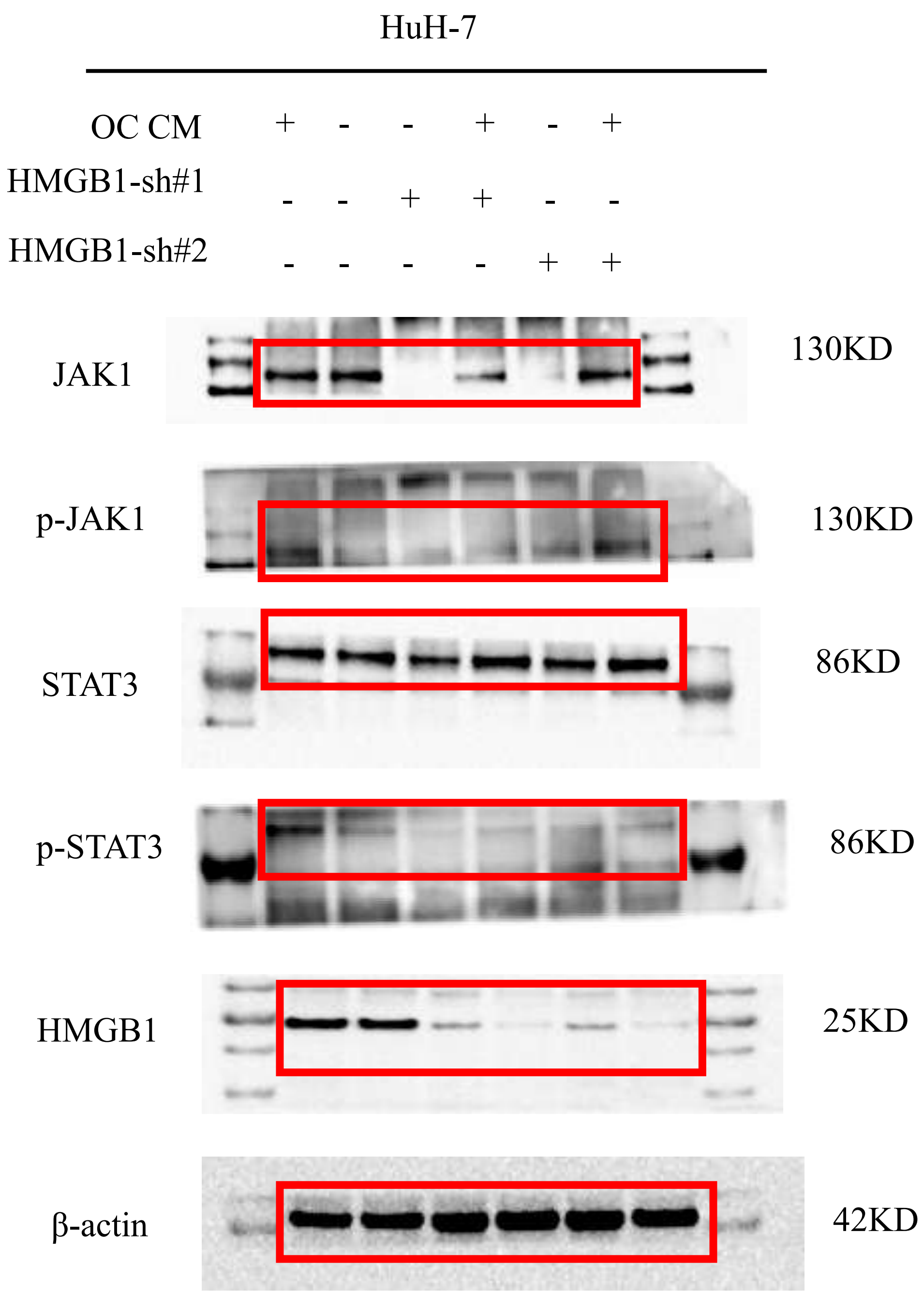
HuH-7
OC CM + - - + - +
HMGB1-sh#1 - - + + - -
HMGB1-sh#2 - - - - + +
JAK1
130KD
p-JAK1
130KD
STAT3
86KD
p-STAT3
86KD
HMGB1
25KD
β-actin
42KD

Fig5C

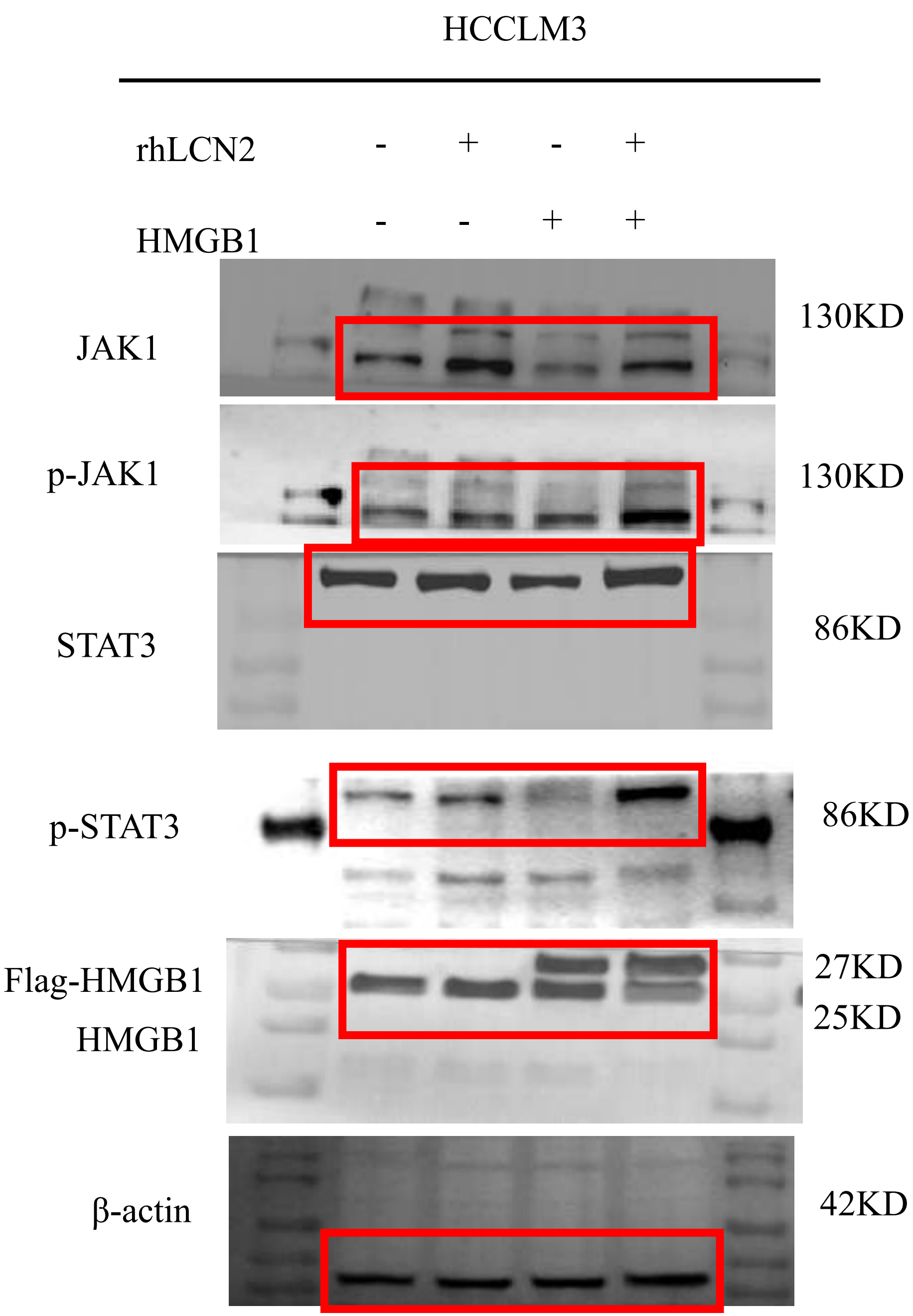
HCCLM3
rhLCN2 - + - +
HMGB1 - - + +
JAK1
130KD
p-JAK1
130KD
STAT3
86KD
p-STAT3
86KD
Flag-HMGB1
HMGB1
27KD
25KD
β-actin
42KD

Fig5C

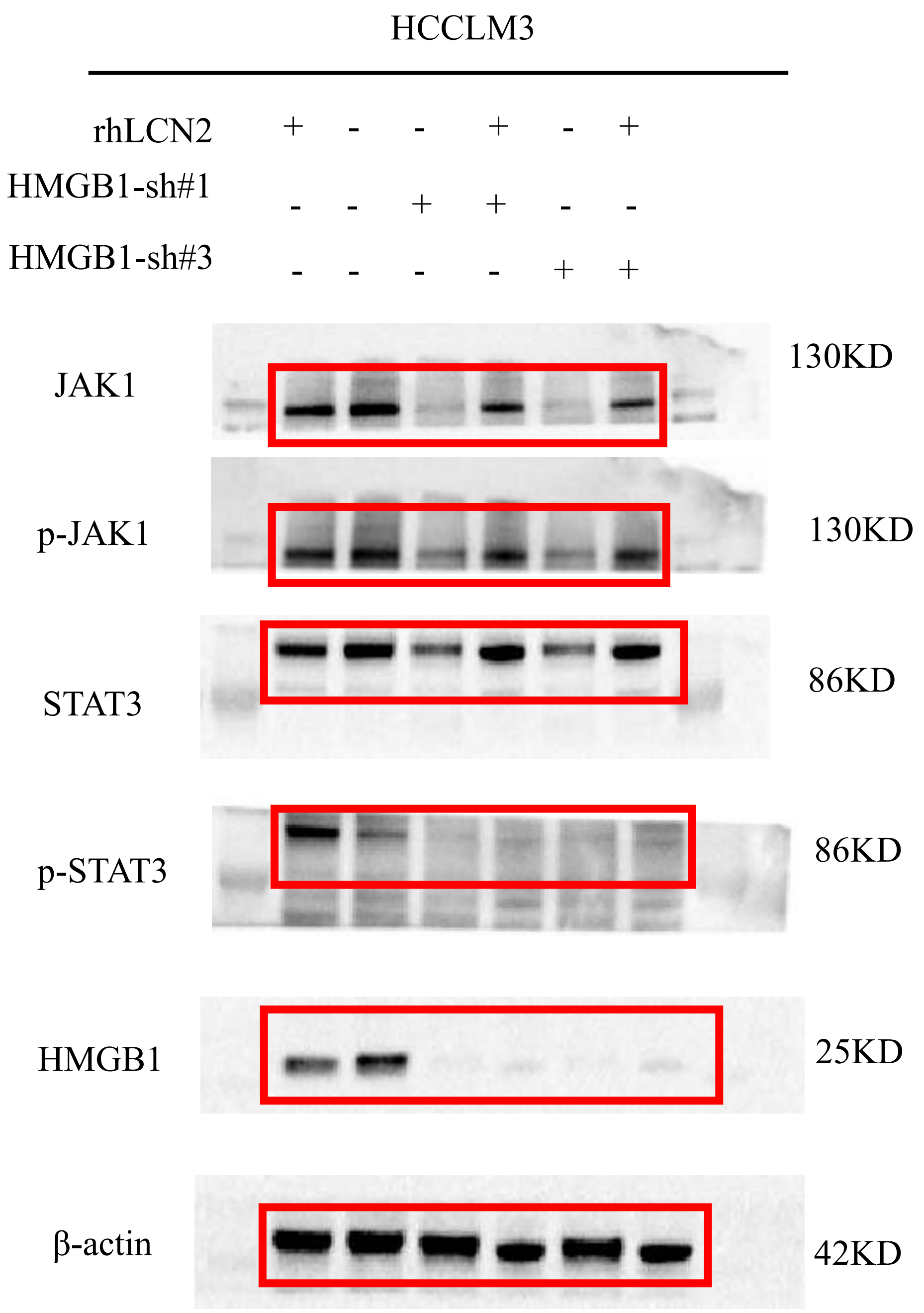
HCCLM3
rhLCN2 + - - + - +
HMGB1-sh#1 - - + + - -
HMGB1-sh#3 - - - - + +
JAK1
130KD
p-JAK1
130KD
STAT3
86KD
p-STAT3
86KD
HMGB1
25KD
β-actin
42KD

Fig5D

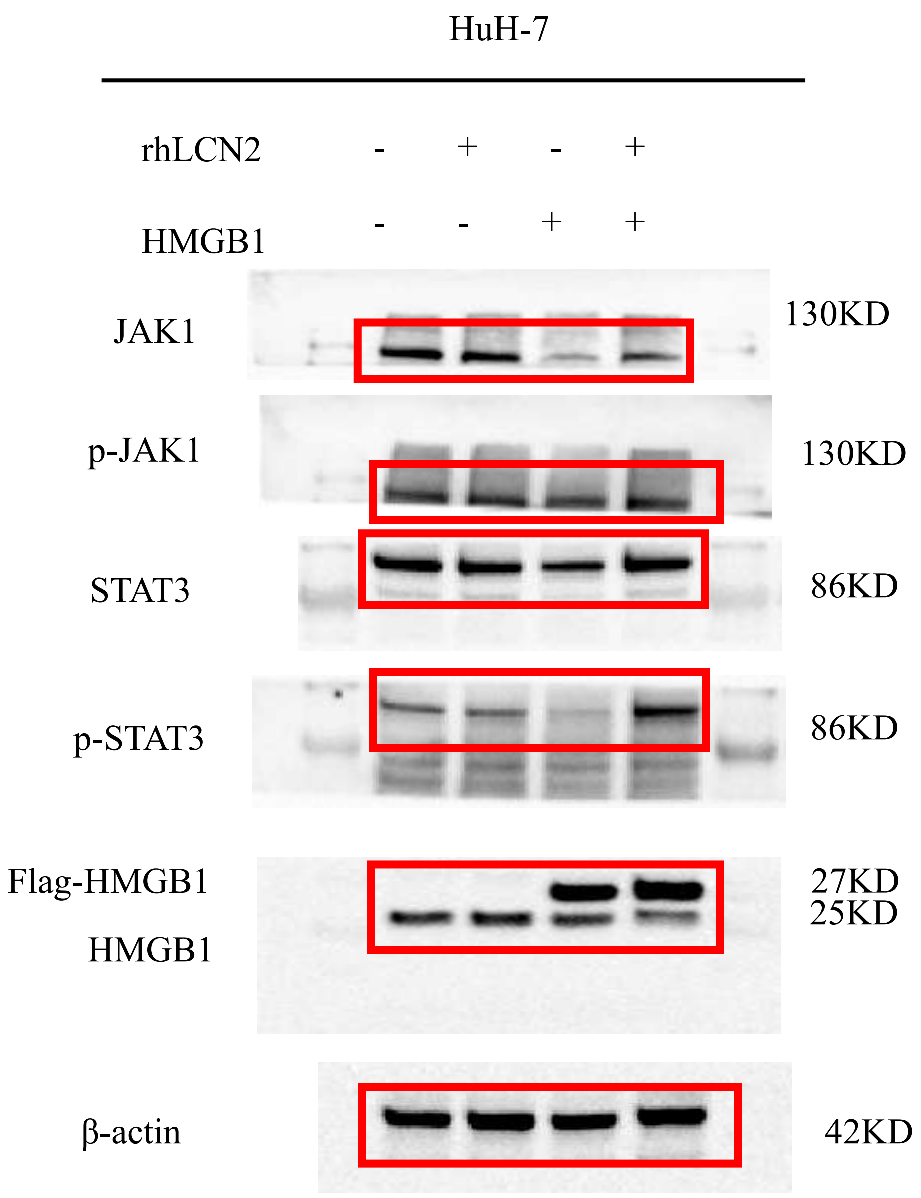
HuH-7
rhLCN2 - + - +
HMGB1 - - + +
JAK1
130KD
p-JAK1
130KD
STAT3
86KD
p-STAT3
86KD
Flag-HMGB1
HMGB1
27KD
25KD
β-actin
42KD

Fig5D

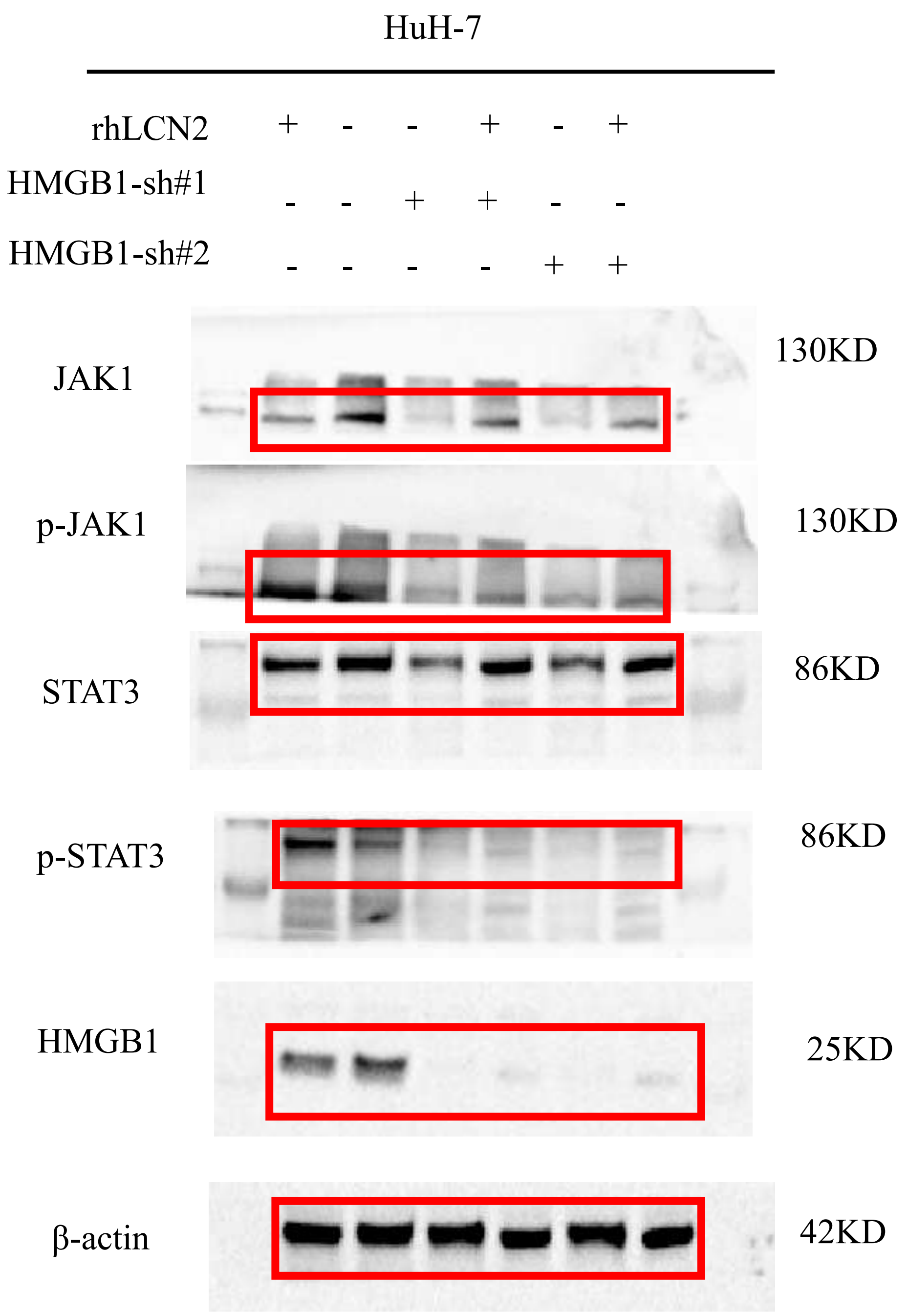
HuH-7
rhLCN2 + - - + - +
HMGB1-sh#1 - - + + - -
HMGB1-sh#2 - - - - + +
JAK1
130KD
p-JAK1
130KD
STAT3
86KD
p-STAT3
86KD
HMGB1
25KD
β-actin
42KD

Fig6A

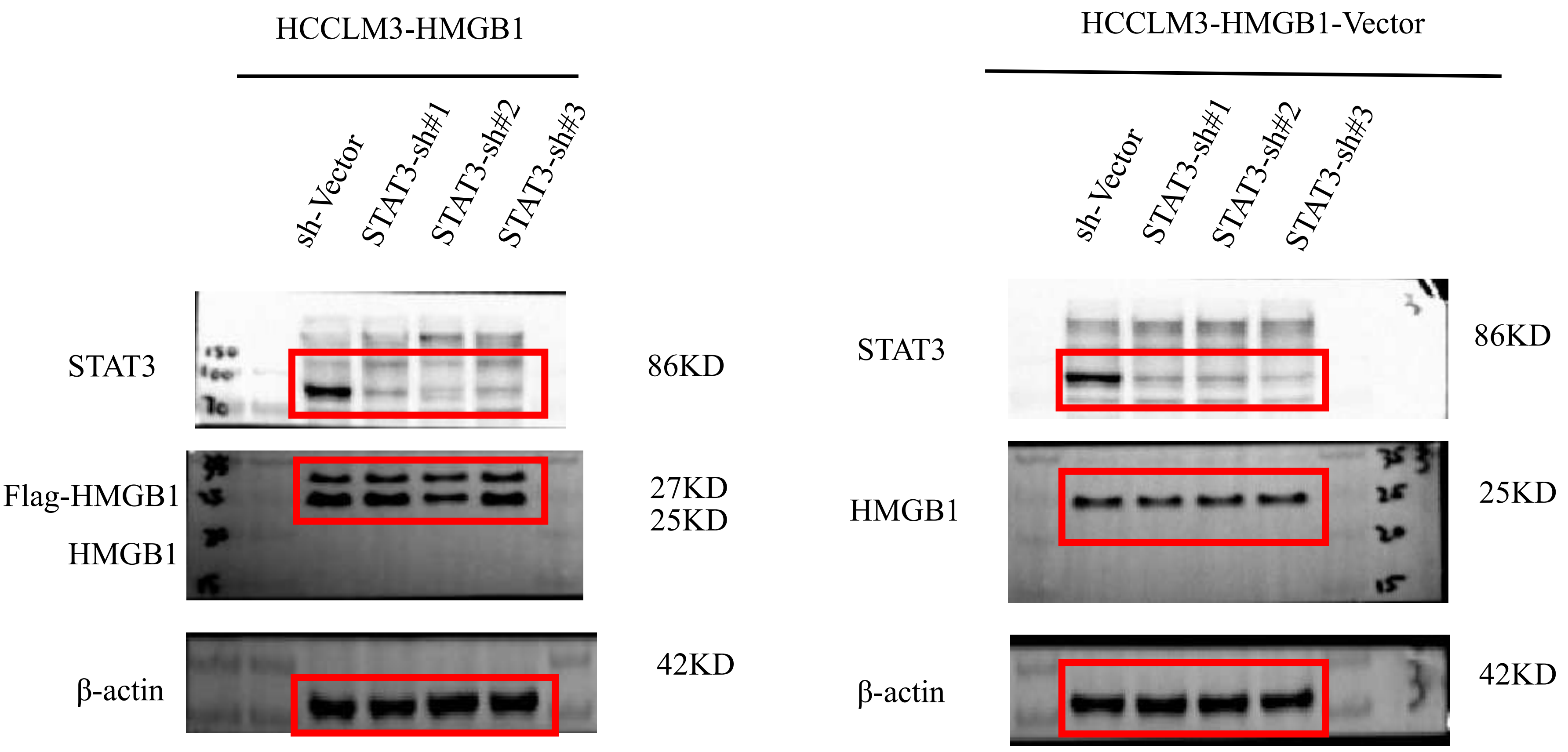
HCCLM3-HMGB1
sh-Vector
STAT3-sh#1
STAT3-sh#2
STAT3-sh#3
STAT3
86KD
Flag-HMGB1
HMGB1
27KD
25KD
β-actin
42KD
HCCLM3-HMGB1-Vector
sh-Vector
STAT3-sh#1
STAT3-sh#2
STAT3-sh#3
STAT3
86KD
HMGB1
25KD
β-actin
42KD

Fig6G

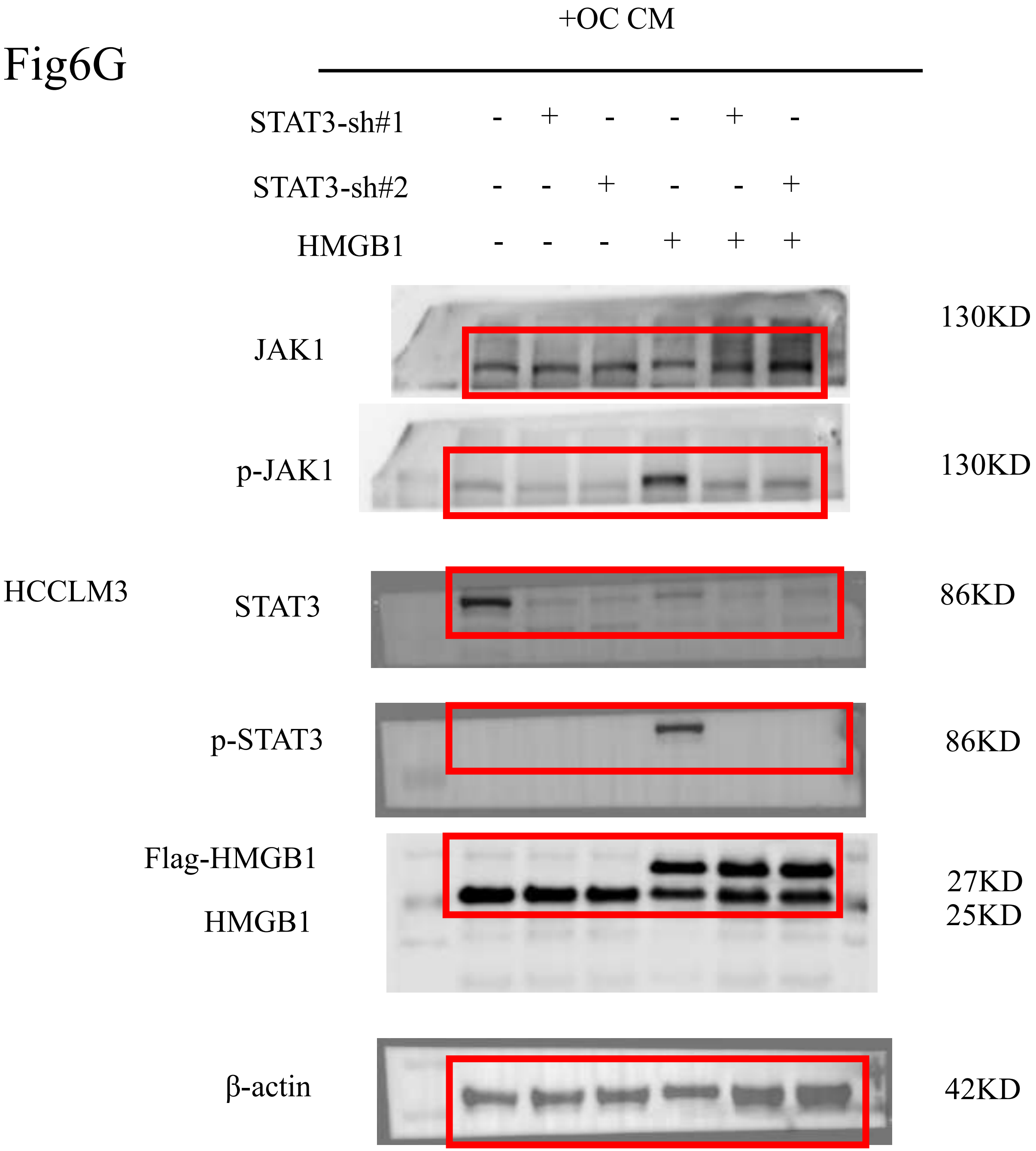
+OC CM
STAT3-sh#1 - + - - + -
STAT3-sh#2 - - + - - +
HMGB1 - - - + + +
HCCLM3
JAK1
p-JAK1
STAT3
p-STAT3
Flag-HMGB1
HMGB1
β-actin
130KD
130KD
86KD
86KD
27KD
25KD
42KD

Fig6G

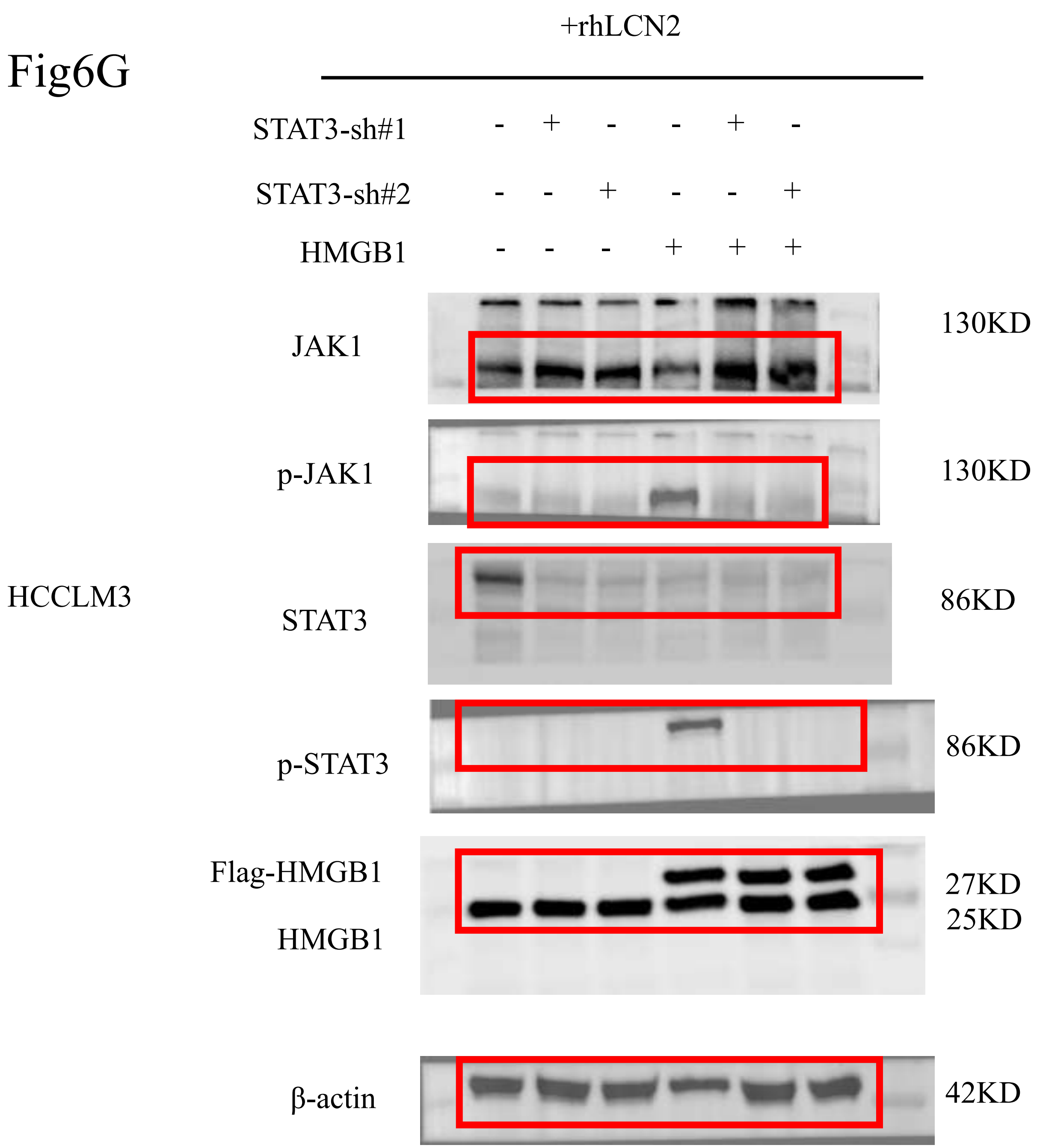
+rhLCN2
STAT3-sh#1 - + - - + -
STAT3-sh#2 - - + - - +
HMGB1 - - - + + +
HCCLM3
JAK1
130KD
p-JAK1
130KD
STAT3
86KD
p-STAT3
86KD
Flag-HMGB1
HMGB1
27KD
25KD
β-actin
42KD

FigS5E

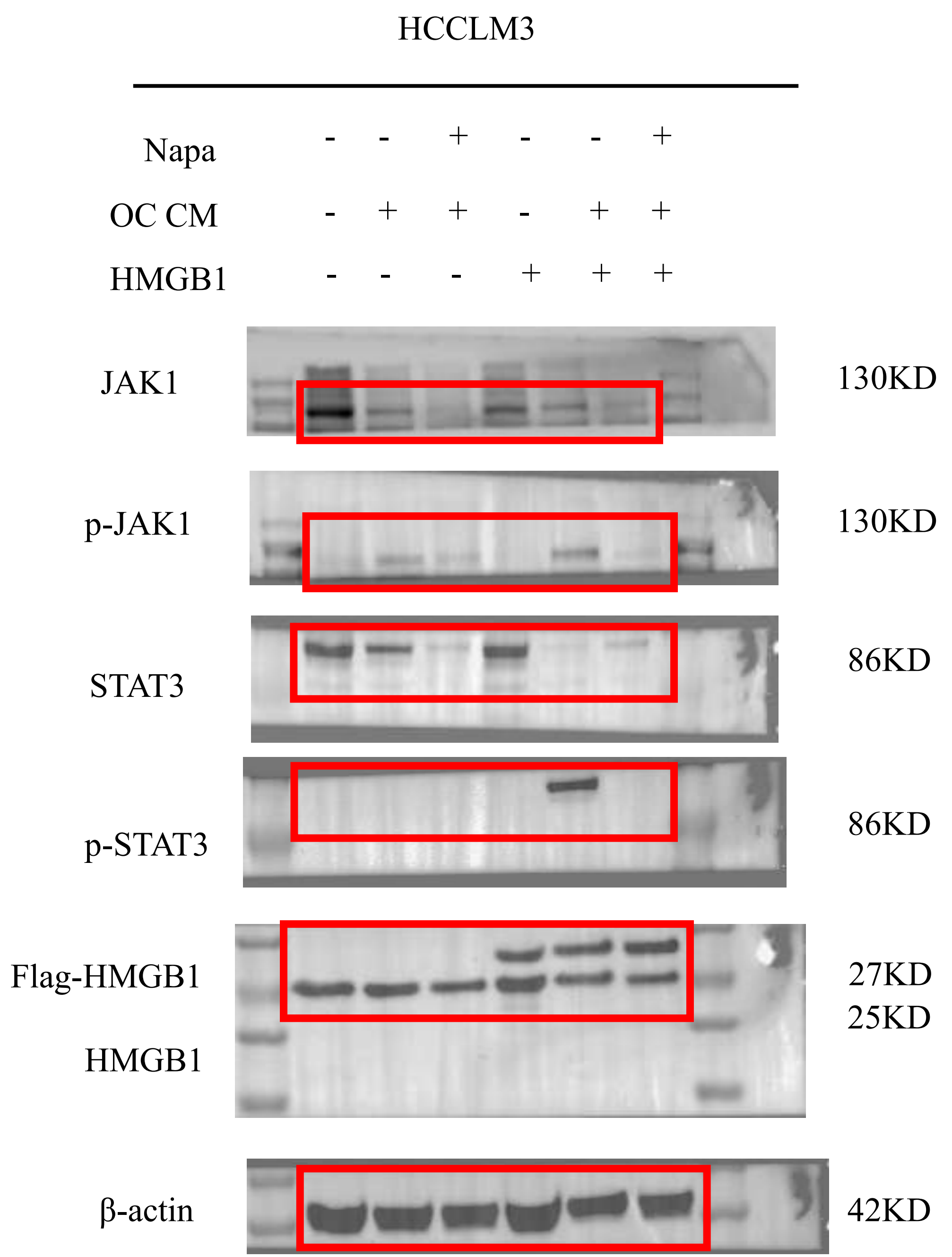
HCCLM3
Napa - - + - - +
OC CM - + + - + +
HMGB1 - - - + + +
JAK1
130KD
p-JAK1
130KD
STAT3
86KD
p-STAT3
86KD
Flag-HMGB1
27KD
25KD
HMGB1
β-actin
42KD

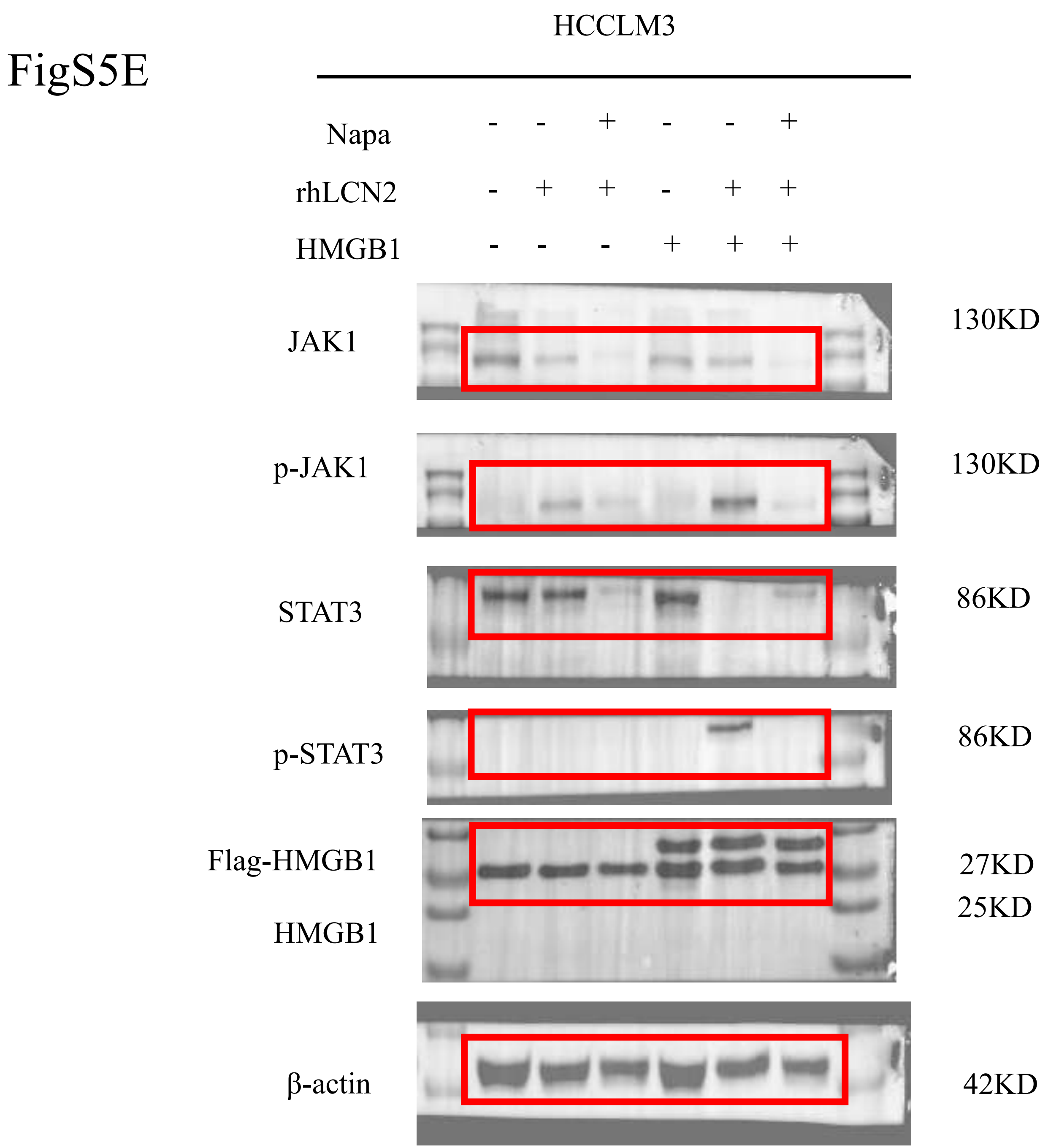
FigS5E
HCCLM3
Napa - - + - - +
rhLCN2 - + + - + +
HMGB1 - - - + + +
JAK1
130KD
p-JAK1
130KD
STAT3
86KD
p-STAT3
86KD
Flag-HMGB1
HMGB1
27KD
25KD
β-actin
42KD

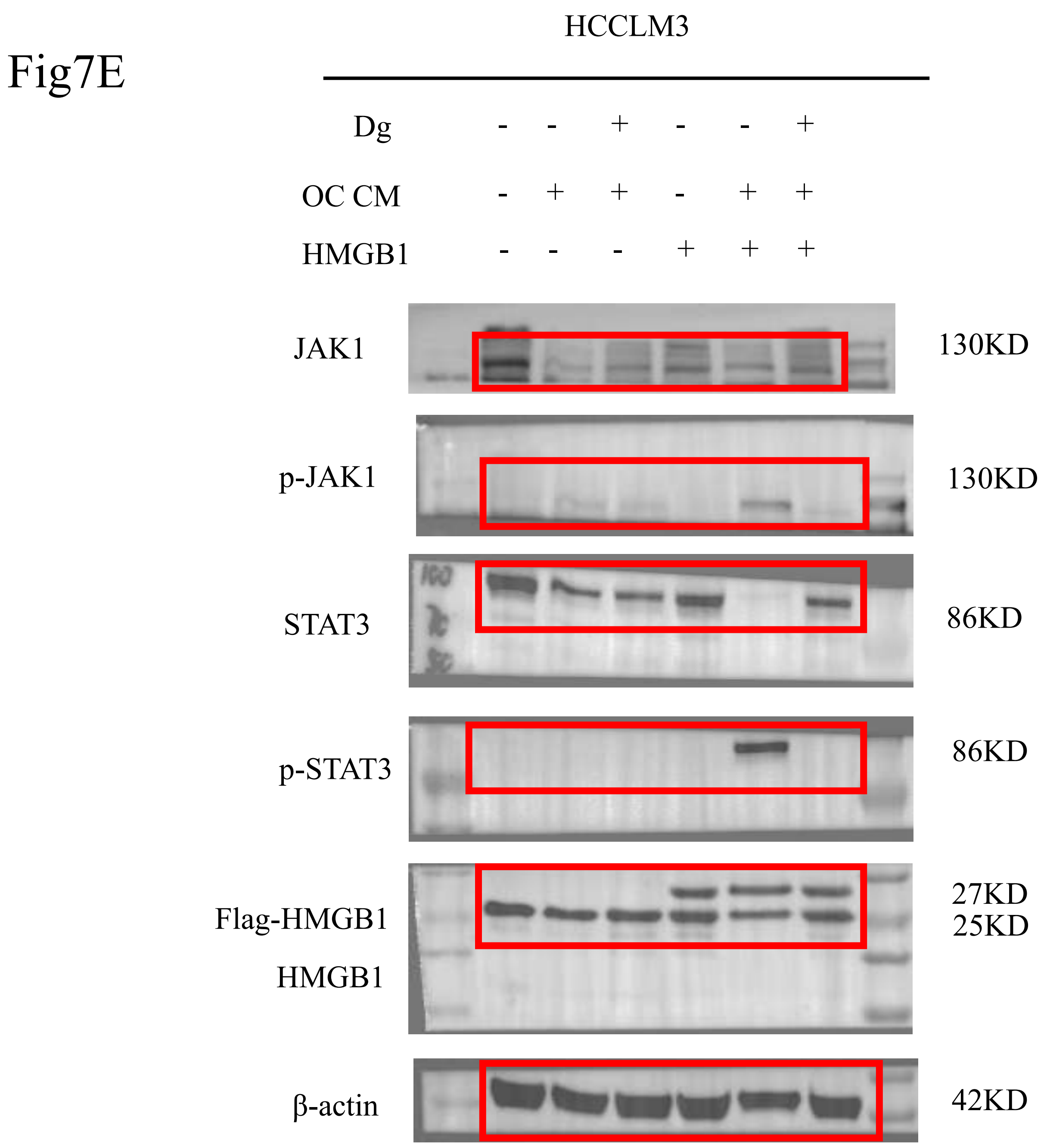
Fig7E
HCCLM3
Dg - - + - - +
OC CM - + + - + +
HMGB1 - - - + + +
JAK1
130KD
p-JAK1
130KD
STAT3
86KD
p-STAT3
86KD
Flag-HMGB1
HMGB1
27KD
25KD
β-actin
42KD

Fig7F

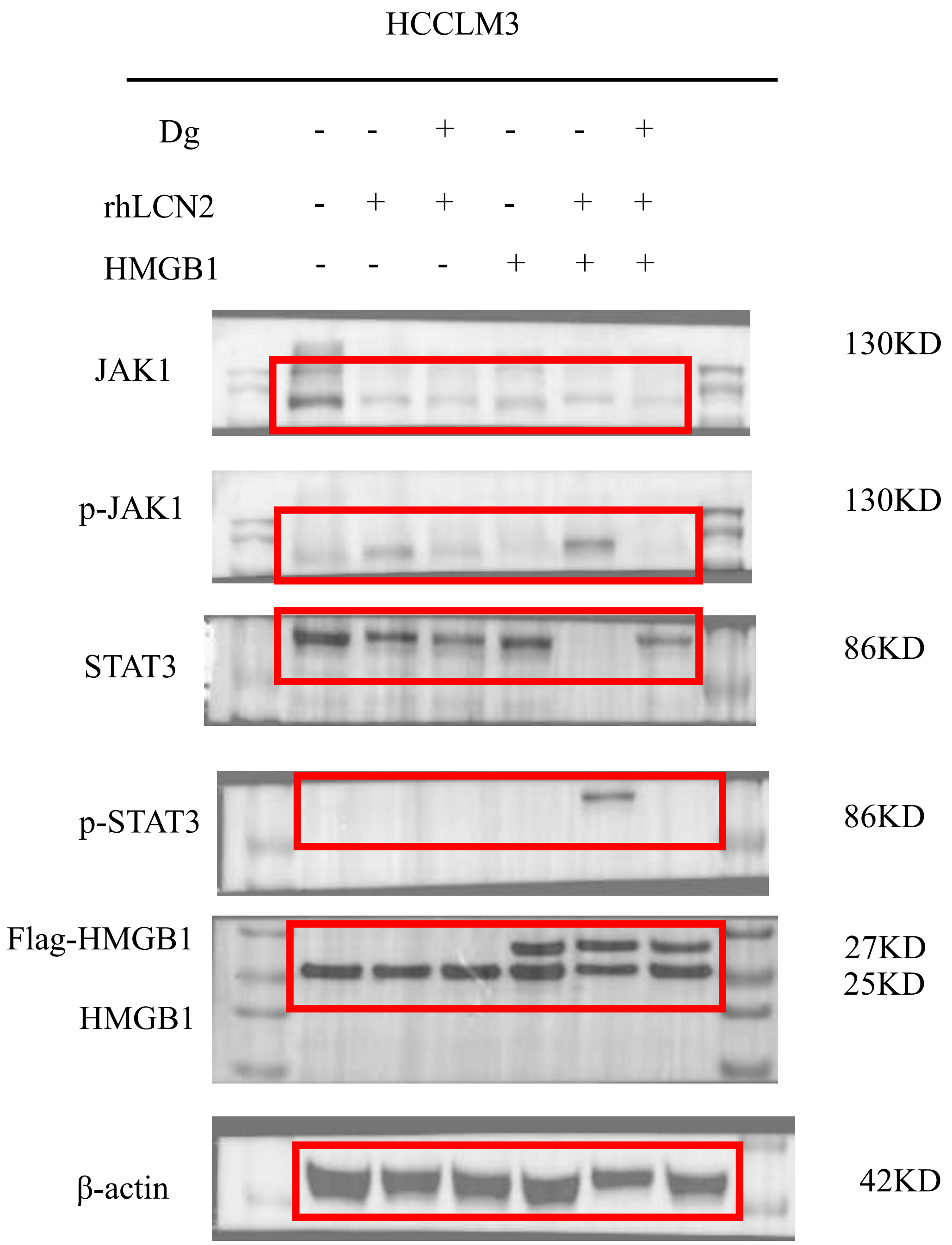
HCCLM3
Dg - - + - - +
rhLCN2 - + + - + +
HMGB1 - - - + + +
JAK1
130KD
p-JAK1
130KD
STAT3
86KD
p-STAT3
86KD
Flag-HMGB1
HMGB1
27KD
25KD
β-actin
42KD
