# Supplementary figures and images for: HMGB1 orchestrates tumor-osteoclast crosstalk to drive bone metastasis in hepatocellular carcinoma
Source: Cell Death Dis. 2025 Oct 7;16(1):712. doi: 10.1038/s41419-025-08037-6 (PMC12504543; doi:10.1038/s41419-025-08037-6)

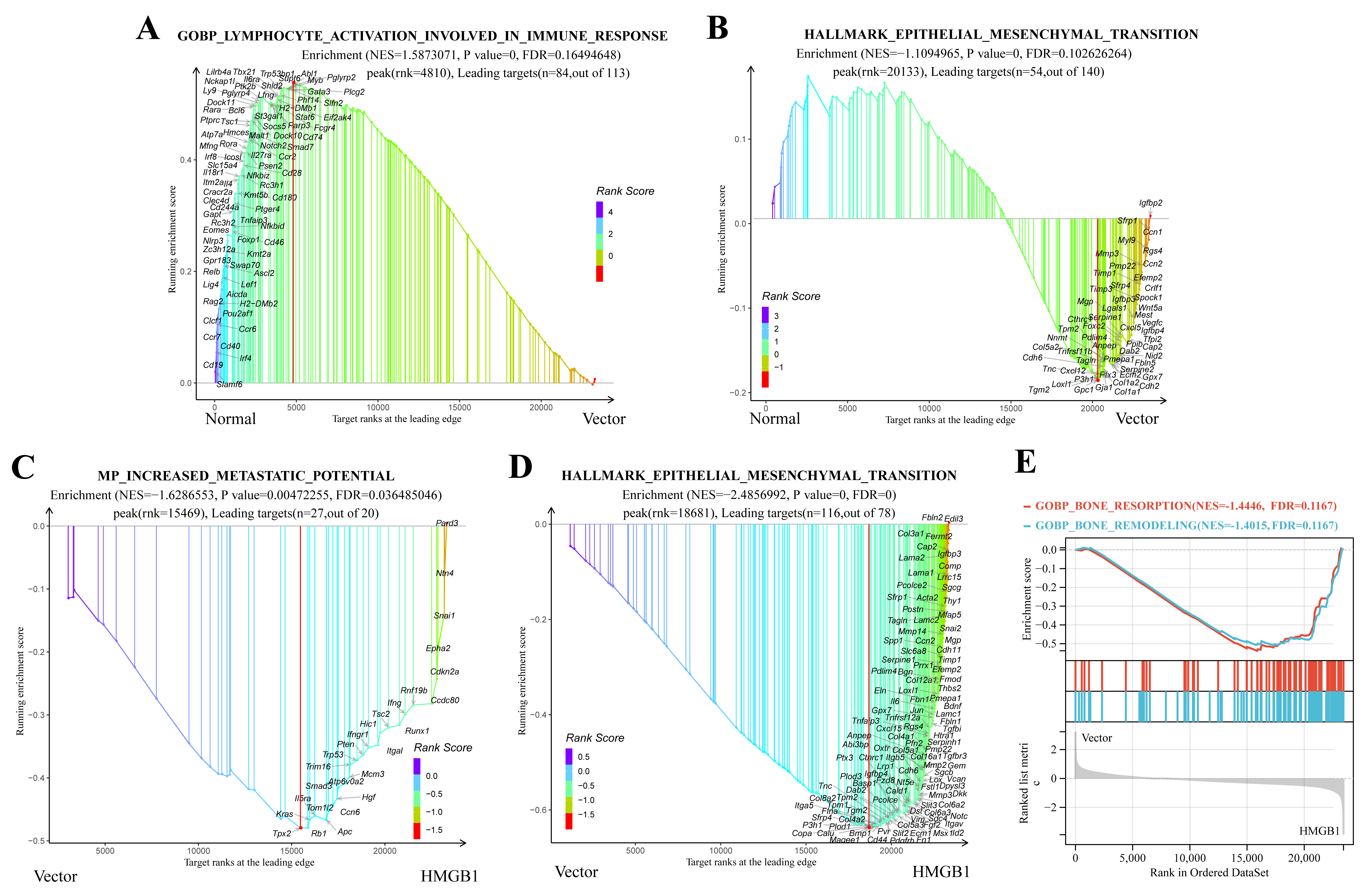

Supplement: Supplementary file 4 — Figure S1 [file 41419_2025_8037_MOESM4_ESM.png]

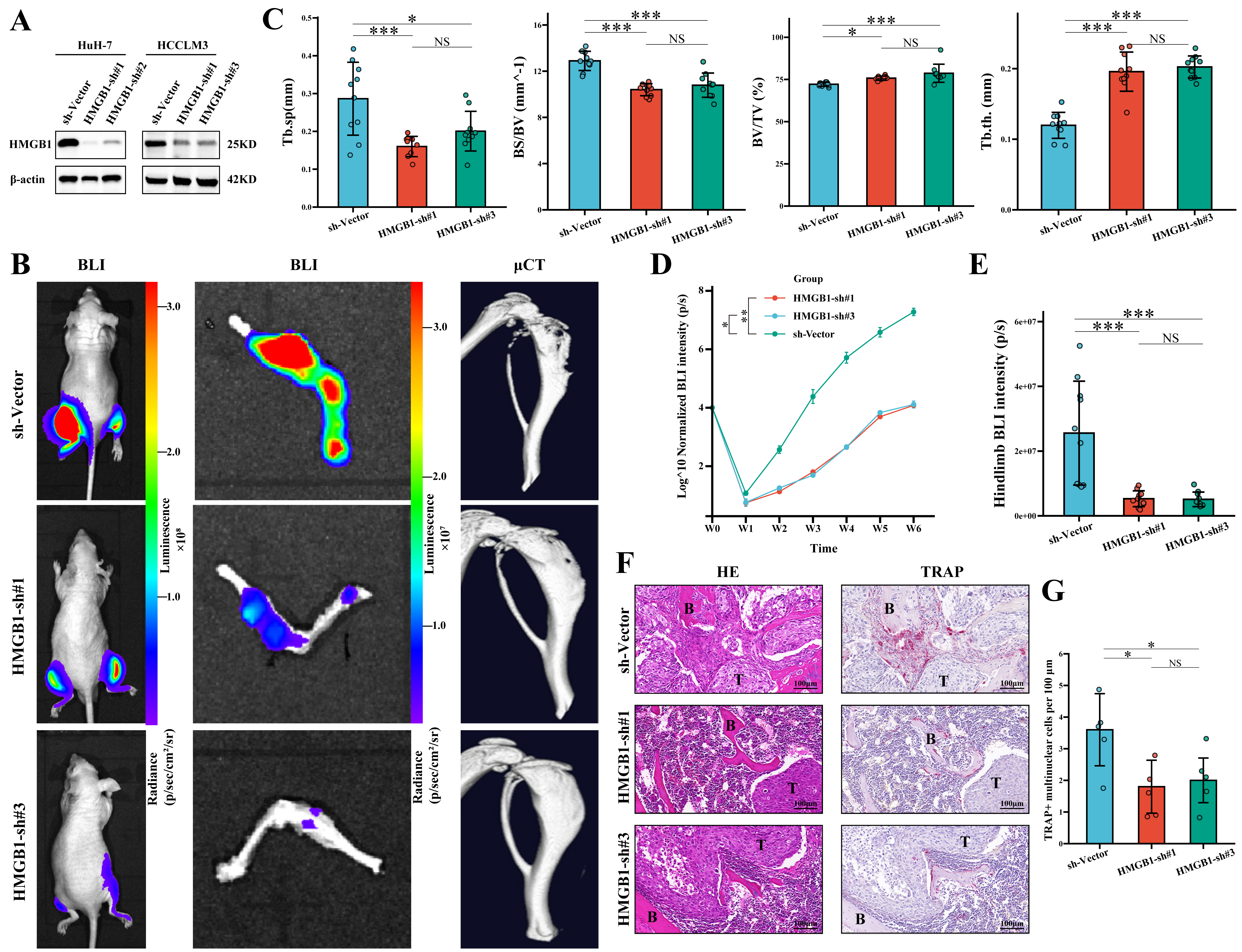

Supplement: Supplementary file 5 — Figure S2 [file 41419_2025_8037_MOESM5_ESM.png]

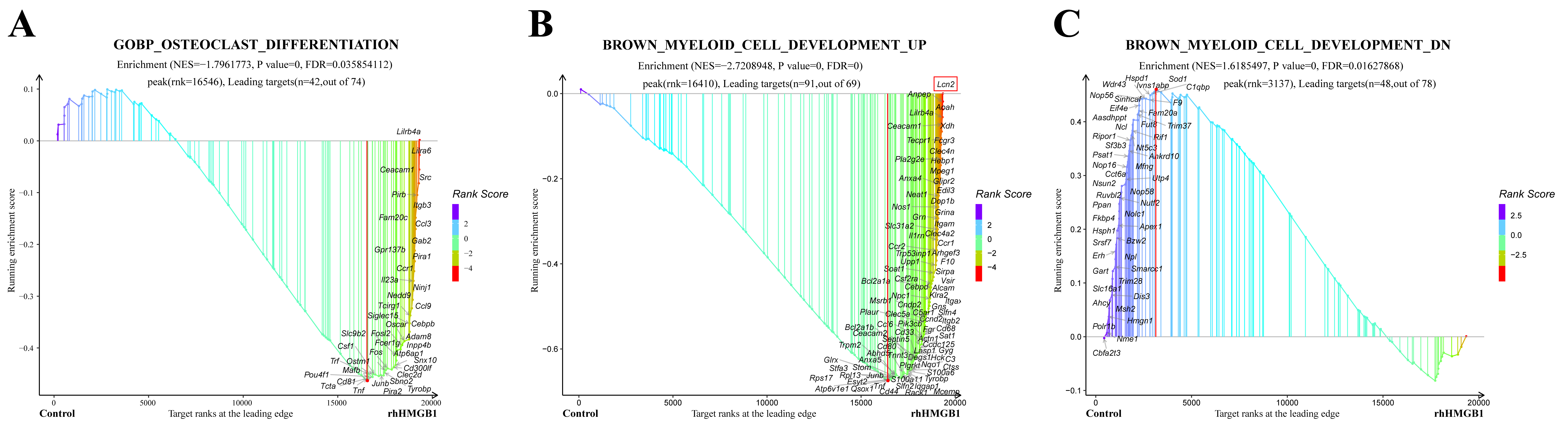

Supplement: Supplementary file 6 — Figure S3 [file 41419_2025_8037_MOESM6_ESM.png]

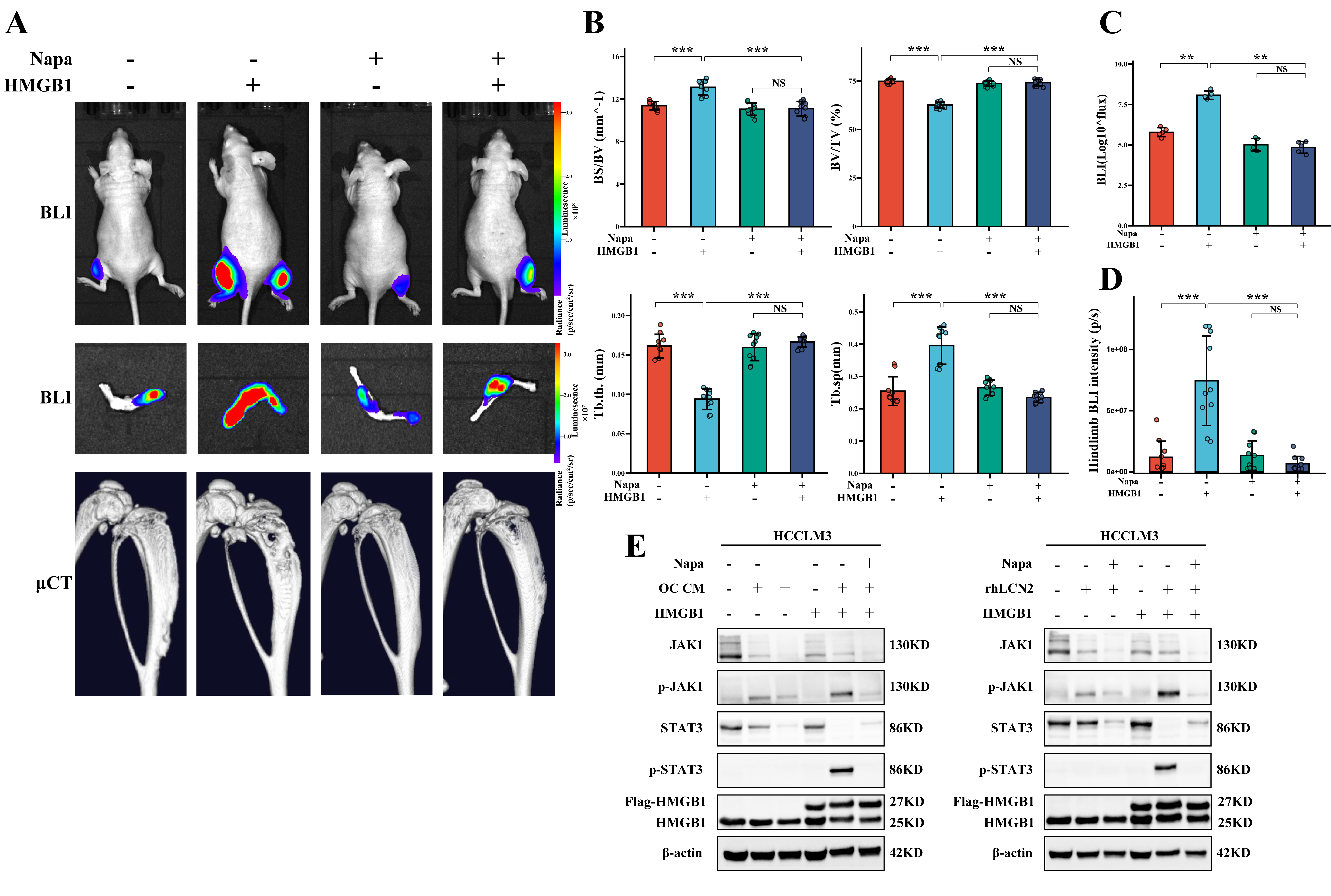

Supplement: Supplementary file 8 — Figure S5 [file 41419_2025_8037_MOESM8_ESM.png]
